# A Scoping Review of Genus *Viscum*: Biological and Chemical Aspects of Alcoholic Extracts

**DOI:** 10.3390/plants12091811

**Published:** 2023-04-28

**Authors:** Michelle Nonato de Oliveira Melo, João Vitor da Costa Batista, Evelyn Maribel Condori Peñaloza, Adriana Passos Oliveira, Rafael Garrett, Stephan Baumgartner, Carla Holandino

**Affiliations:** 1 Multidisciplinary Laboratory of Pharmaceutical Sciences, Faculty of Pharmacy, Universidade Federal do Rio de Janeiro, Rio de Janeiro 21941-902, Brazil; 2 Metabolomics Laboratory, Chemistry Institute, Universidade Federal do Rio de Janeiro, Rio de Janeiro 21941-598, Brazil; 3 Society for Cancer Research, Hiscia Institute, Kirschweg 9, 4144 Arlesheim, Switzerland; 4 Department of Pharmaceutical Sciences, Division of Pharmaceutical Technology, University of Basel, 4056 Basel, Switzerland; 5 Institute of Integrative Medicine, University of Witten/Herdecke, Gerhard-Kienle-Weg 4, 58313 Herdecke, Germany; 6 Institute of Complementary and Integrative Medicine, University of Bern, Freiburgstrasse 46, 3010 Bern, Switzerland

**Keywords:** *Viscum*, mistletoe, alcoholic extract, biological activity, chemistry

## Abstract

The genus *Viscum* comprises a large number of semi-parasitic shrubs popularly known as Mistletoe. The *Viscum* species grow in many countries of Europe, Africa and Asia with different popular uses in ornamentation, foods and medicine. Many studies about *Viscum* have been done over the last years focusing on biological activities and chemical composition of the aqueous extracts, mainly related to anthroposophical medicines. However, it is known that non-aqueous preparations, as alcoholic extracts, have demonstrated different biological activities that are species—and host tree—dependent. Considering the potential of these alcoholic extracts, a scoping review was conducted using data from three online databases: PubMed, Scopus and Embase. Inclusion criteria consisted of the in vitro, in vivo, ex vivo, clinical and chemical studies of alcoholic extracts from *Viscum* species. The present review summarized 124 original publications about fourteen *Viscum* species. *Viscum album*, *Viscum articulatum* and *Viscum coloratum* were the main studied species. Alcoholic extracts demonstrated hypotensive, anticancer, antimicrobial, analgesic and anti-inflammatory capabilities, among other biological activities. Flavonoids, phenolic acids and terpenoids represented 48%, 24% and 11% of the total identified compounds, respectively. This review contributes to the knowledge of alcoholic preparations of the *Viscum* species and points out the lack of clinical studies concerning these different extracts.

## 1. Introduction

The genus *Viscum* includes 100–150 species distributed between tropical and temperate regions in Europe, Africa and Asia [1,2]. Mistletoe, as it is popularly known, is an evergreen hemiparasite shrub that grows on different host trees, which reflects important features in the biological activities of these species. A root-like organ called haustorium intimately connects the mistletoe to the host trees, and this structure makes it possible for these plants to absorb solutes and water from their hosts. Species of this genus produce a fruit called berry, which has different colors, depending on the *Viscum* sp., and this characteristic can help differentiate their origin [3]. 

The use of European mistletoe in medicine is ancient. Hippocrates (460–377 BC) described this plant to treat diseases in the spleen and complaints associated with menstruation [3]. In 150 AD, Platonist Celsus also reported the use of mistletoe against swellings and tumors. In the 16th century, mistletoe appeared in many European writings describing its medicinal use against epilepsy, bone fractures and kidney and spleen diseases [3]. Other applications of mistletoe, such as edema and “weakness of the heart”, were reported in the 18th century, which is described in Boericke’s homeopathic Materia Medica [4]. More recently, in the beginning of the 20th century, the famous European mistletoe—*Viscum album*—was introduced by Rudolf Steiner and Ita Wegman as an anthroposophical remedy for cancer treatment [5].

The interest in the biological potential of *Viscum* has increased over the last years, and many studies have demonstrated different mechanisms of action and chemical composition of these vegetal species. Despite the vast number of species of this genus, most of the published studies have been carried out mainly with *Viscum album*, *Viscum coloratum* and *Viscum articulatum* [6]. It is known that chemical composition and biological activity can be influenced by the *Viscum* species and subspecies, its host tree and the extracting solvent [7,8,9]. Shaller and co-workers showed that *Viscum album* acid aqueous extracts of three subspecies from nine different host trees presented a host-dependency in their content of viscotoxins [10]. Loef and Walach also demonstrated that cancer patients’ quality of life was benefited by the aqueous *Viscum album* extracts, but the clinical response had a varied considering the specific host tree [11]. 

Besides the extensive publications with aqueous extracts in the last decades, other extractive forms, such as alcoholic preparations of the *Viscum* species, have shown biological potential, which is also species—and host tree—dependent. These alcoholic extracts have shown antimicrobial [12], cytotoxic [13], anti-inflammatory [14] and antihypertensive activities [15], among other activities. However, gaps in the actual knowledge were identified in respect to a systematic evaluation of the in vitro, in vivo, clinical and chemical composition studies that can support the uses of this alcoholic extraction. In this sense, the aim of this review was to systematically organize and summarize the important research progress made in the use of the alcoholic *Viscum* extracts, discussing and highlighting the different approaches applied to the genus *Viscum*, elucidating the targets and mechanisms of action of these alcoholic extracts and guiding future research and potential clinical applications.

## 2. Results and Discussions

**Figure 1:**
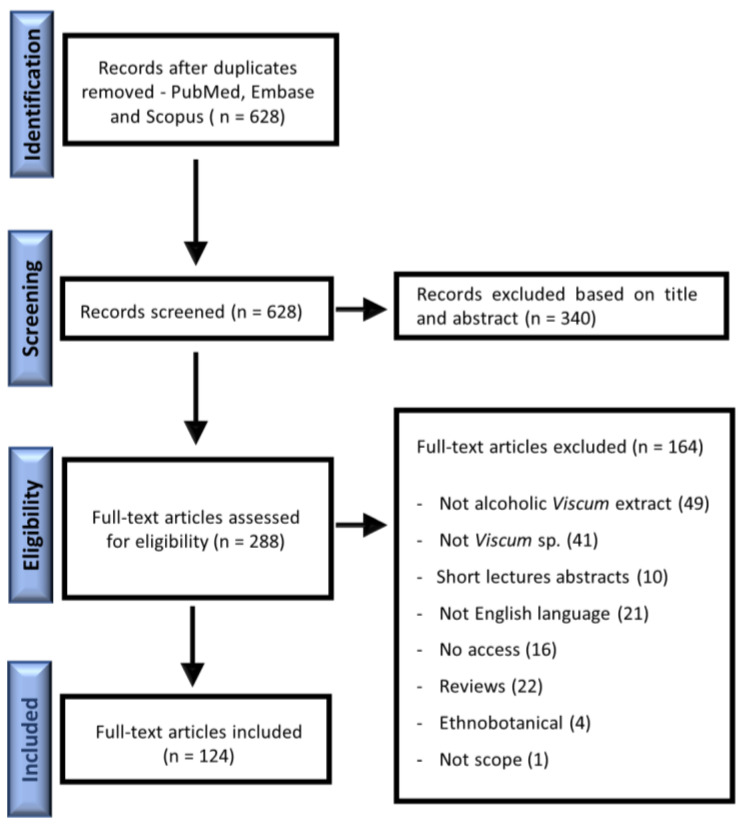
Flow chart of study identification, inclusion and exclusion criteria.

The genus *Viscum* has received considerable interest due to its popularity, mainly the *V. album* species. Many studies have been performed with the genus *Viscum* in the last decades, but most of them have focused on aqueous extracts. The present review evaluated only alcoholic extractions considering their potential pharmacological activities. The identification step allowed us to have 628 hits according to the keywords. After duplication exclusion and abstract evaluation, 288 publications were fully evaluated. In this step, 164 publications were excluded, and 124 original publications were included in this review, as described in Figure 1. 

**Figure 2:**
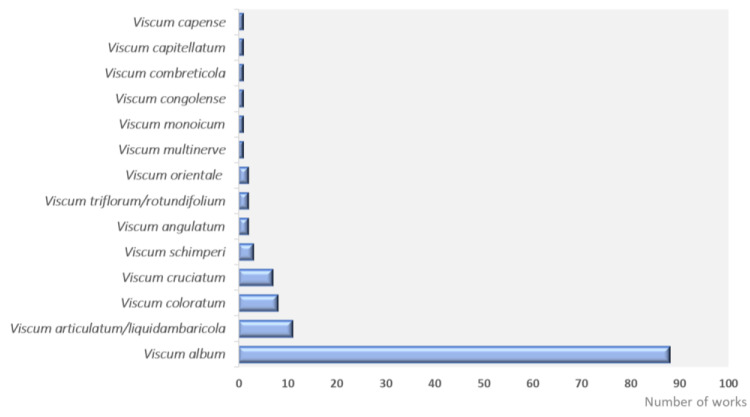
Species of the genus *Viscum* reported in the included studies of this review.

Fourteen species were identified in this review (Figure 2), and the most cited species were *V. album* L. (87 studies), *V. articulatum* (10 studies) and *V. coloratum* (8 studies). However, according to the *International Plant Names Index* (IPNI) [16] and *WFO Plant List* [17], some names for *Viscum* sp. considered by authors are synonymous, such as *V. triflorum* DC and *V. rotundifolium* Bory*, V. coloratum* Nakai which is another name of the *V. album* var. *colaratum* and *V. articulatum* that can be found as *V. liquidambaricola* Hayata. A similar pattern in relation to the main species of the genus *Viscum* studied worldwide was also found by Song et al. [6] but with different proportions *V. coloratum* > *V. album* > *V. articulatum* [6]. The authors did not delimit the solvent extractor and included different extractive forms.

**Figure 3:**
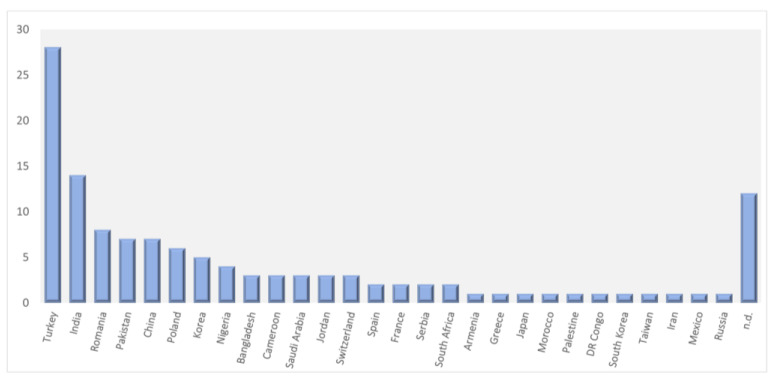
Origin of the *Viscum* sp. in the included studies by number of works; n.d.—origin not determined in the publication.

The main harvesting regions of *Viscum* sp. were Turkey, India, Romania, Pakistan, China, and Poland, which corresponded to 56% of included studies (Figure 3). It is noteworthy that twelve studies did not present the *Viscum* sp.’s origin. On the other hand, concerning the parts of the plant, 83% of the authors reported the use of their fruits, leaves, stems or whole plant. Regarding the solvent, approximately 50% of the studies used methanol and 48% ethanol (Table 1).

The correct description of the species, subspecies, site of harvesting, as well as the extract solvent and the standardization of the extraction methods are crucial for the quality evaluation of mistletoe herbal products and its biological activities [7]. Additionally, identification errors or uncertainty regarding the plant origin can lead to serious safety problems [18]. In this scenario, the present work highlights a significant number of *Viscum* species that have not yet been well studied, which opens to new and more innovative research in this area.

**Figure 4:**
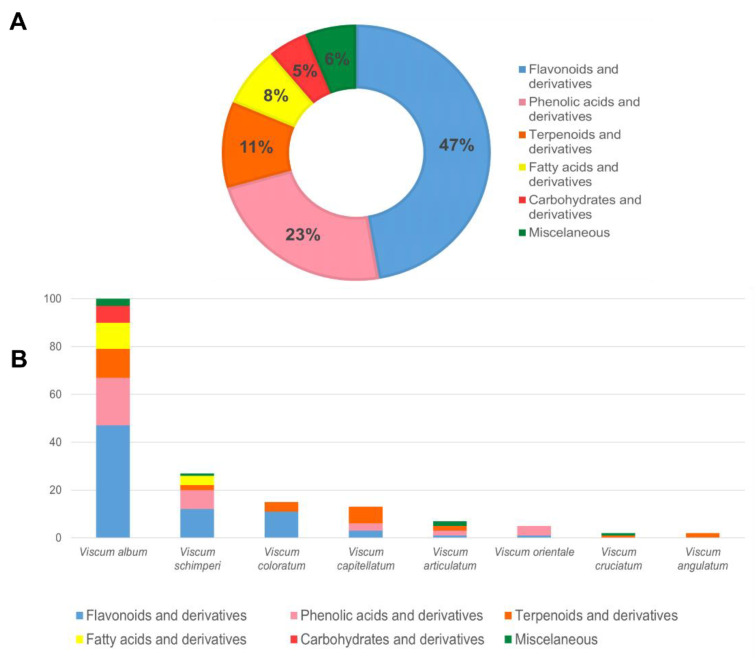
Percentage of the chemical classes found in this review (**A**) and chemical classes by *Viscum* sp. (**B**).

Alcoholic solvents are a good strategy for phenolic compounds extraction [19,20], and our results demonstrated that approximately 70% of the chemical compounds identified by authors were flavonoids and phenolic acids (Figure 4). Maceration accounts to 31% of the included studies as the method for plant extraction. This process has been used over the years considering some aspects such as simplicity and low cost of the operation. It was possible to find articles in which maceration was used as an extraction methodology from 1998 [21] to 2022 [22,23]. However, this method presents some disadvantages such as long extraction time and low extraction efficiency [24]. The second most common approach was the use of Soxhlet apparatus that is characterized by shorter extraction time and less solvent consumption when compared to maceration or percolation processes. However, the use of high temperature in this methodology increases compounds’ thermal degradation [24]. Therefore, even though the same species was extracted using the same method, the solvent was not always the same, leading to difficulties for comparison in this review.

**Table 1:** General data of included articles with alcoholic *Viscum* sp. extracts.

Species	Subspecies/Var.	Parts Used	Extractive Solvent	Extractive Method	Harvest Month	Authors
*Viscum album*	subsp. *album*	Leaves, stems, berries	Methanol 80%	Bead mill (30 Hz)	September	[7]
*Viscum album*	n.d.	Leaves and twigs	Ethanol, methanol	Maceration at room temperature	n.d.	[12]
*Viscum album*	n.d.	n.d.	Methanol	Soxhlet extraction	July	[13]
*Viscum coloratum*	n.d.	n.d.	Ethanol 70%	Boiling	n.d.	[14]
*Viscum album*	n.d.	Stems	Ethanol	Maceration at room temperature	June	[15]
*Viscum album*	n.d.	Leaves and stems	Ethanol	Maceration	n.d.	[21]
*Viscum album*	subsp. *abietis*	Whole plant	Ethanol 70%	Room temperature maceration	January	[22]
*Viscum album*	n.d.	Leaves	Methanol	Maceration at room temperature	n.d.	[25]
*Viscum album*	n.d.	Leaves and stems	Ethanol	Percolation at room temperature	n.d.	[26]
*Viscum album*	n.d.	Leaves and flowers	Ethanol 95%	Cold maceration	October	[27]
*Viscum album*	n.d.	Leaves	Ethanol 96%	High temperature maceration	April	[28]
*Viscum album*	n.d.	Aerial parts	Methanol	Soxhlet extraction	n.d.	[29]
*Viscum album*	n.d.	Stem, leaves and fruits	Methanol	Maceration	April	[30]
*Viscum album*	var. *coloratum*	Stems	Ethanol 70%	Maceration	n.d.	[31]
*Viscum album*	subsp. *album*	Leaves	Ethanol 50%	Waring blender at room temperature	February	[32]
*Viscum album*	subsp. *austriacum*	Leaves, fruits and bodies	Ethanol 80%	Intermittent shaking	September, October	[33]
*Viscum album*	n.d.	Leaves, stems and flowers	Ethanol 95%	Maceration	March, April	[34]
*Viscum album*	n.d.	n.d.	Ethanol 45%	Maceration	n.d.	[35]
*Viscum album*	subsp. *album*	Leaves	Methanol	Maceration	July	[36]
*Viscum album*	n.d.	Leaves	Methanol	Maceration	September	[37]
*Viscum album*	n.d.	Leaves and stems	Ethanol/Methanol	Maceration	n.d	[38]
*Viscum album*	subsp. *album*, *austriacum*	Fruits	Methanol 80%	Ultrasound assisted maceration at room temperature	n.d.	[39]
*Viscum album*	n.d.	Leaves and branches	Ethanol 95%, methanol 5%	Magnetic stirring	March	[40]
*Viscum album*	n.d.	Leaves and stems	Ethanol	Cold chamber with circular agitation	February, November	[41]
*Viscum album*	n.d.	Fruits	Ethanol 80%	Ultrasonic bath	May	[42]
*Viscum album*	subsp. *album*, *austriacum*, *abietis*	n.d.	Ethanol 80%	Room temperature	April, June	[43]
*Viscum album*	subsp. *album*	Herbaceous parts	Methanol	Maceration at room temperature	September	[44]
*Viscum album*	n.d.	Whole plant	Methanol 80%	n.d.	August	[45]
*Viscum album*	subsp. *album*, *austriacum*	Aerial parts	Ethanol 80%	Maceration	n.d.	[46]
*Viscum album*	n.d.	Whole plant	Ethanol	Successive extraction	July, September	[47]
*Viscum album*	n.d.	Aerial parts	Ethanol 70%	Sonication on an ice-bath	n.d.	[48]
*Viscum album*	var. *coloratum*	n.d.	Ethanol 25%, 50%, 75%, 100%	Microwave power extraction	n.d.	[49]
*Viscum album*	n.d.	n.d.	Methanol	Soxhlet extraction	n.d.	[50]
*Viscum album*	n.d.	Leaves	Ethanol 80%	Maceration at room temperature	n.d.	[51]
*Viscum album*	var. *coloratum*	n.d.	Ethanol 70%	High temperature (70 °C)	January	[52]
*Viscum album*	subsp. *album*, *abietis*, *austriacum*	Whole plant	Ethanol 96%	Room temperature	n.d.	[53]
*Viscum album*	subsp. *album*	Whole plant	Ethanol 90%	Room temperature	n.d.	[54]
*Viscum album*	n.d.	n.d.	Ethanol 90%	Cold maceration	November, December	[55]
*Viscum album*	n.d.	n.d.	Ethanol 90%	Maceration	n.d.	[56]
*Viscum album*	n.d.	Aerial parts	Methanol, butanol	Soxhlet extraction	September	[57]
*Viscum album*	subsp. *album*	Leaves	Methanol 80%	Soxhlet extraction	March	[58]
*Viscum album*	n.d.	Leaves	Methanol 80%	Soxhlet extraction	March	[59]
*Viscum album*	var. *coloratum*	Whole plant	Methanol	Cold maceration	March	[60]
*Viscum album*	subsp. *album*	Leaves	Methanol 80%	Percolation	March	[61]
*Viscum album*	subsp. *album*, *abietis*, *austriacum*	Leaves and stems	Ethanol, butanol	n.d.	April, June	[62]
*Viscum album*	subsp. *album*	Leaves and stems	Ethanol 80%	Room temperature	June	[63]
*Viscum album*	subsp. *album*	Leaves	Methanol 80%	Maceration	n.d.	[64]
*Viscum album*	n.d.	Fruit	Methanol	Maceration	November	[65]
*Viscum album*	n.d.	Leaves and fruits	Ethanol 94.7–95.2% *v*/*v*	Maceration at room temperature	n.d.	[66]
*Viscum album*	n.d.	Leaves	Methanol	Warring blender	n.d.	[67]
*Viscum album*	n.d.	Leaves	Methanol	Maceration	n.d.	[68]
*Viscum album*	n.d.	Aerial parts	Ethanol 60%	High temperature maceration	n.d.	[69]
*Viscum album*	subsp. *abietis*	Leaves, stems	Methanol and ethanol 50%, 80% and 100%	Maceration, reflux, ultrasonic extraction	n.d.	[70]
*Viscum album*	n.d.	Leaves and stems	Methanol	Cold maceration	December	[71]
*Viscum album*	n.d.	Leaves and stems	Ethanol 98%	Ultra-Turrax	December, May, July	[72]
*Viscum album*	n.d.	Leaves and twigs	Methanol 80, 96%	Soxhlet extraction	n.d.	[73]
*Viscum album*	n.d.	n.d.	Methanol	Room temperature	n.d.	[74]
*Viscum album*	n.d.	Stem, leaves	Ethanol 95%	Reflux	June	[75]
*Viscum album*	subsp*. album*	Leaves	Methanol	Maceration in an incubatory shaker	February, July	[76]
*Viscum album*	subsp*. album*, *austriacum*	Leaves and stems	Ethanol 80%, methanol	Maceration	April, May, June	[77]
*Viscum album*	n.d.	n.d.	Ethanol 96%	Maceration at room temperature	n.d.	[78]
*Viscum album*	n.d.	Aerial parts	Methanol	Soxhlet extraction	September	[79]
*Viscum album*	var*. coloratum*	Leaves and twigs	Methanol	Successive extraction	n.d.	[80]
*Viscum album*	subsp*. album*, *abietis*	Leaves	Methanol	Rotary incubator	July	[81]
*Viscum album*	n.d.	Leaves and twigs	Methanol	Percolation	n.d.	[82]
*Viscum album*	n.d.	Leaves, stems	Ethanol 70%	Maceration	July	[83]
*Viscum album*	subsp. *album*, *abietis*, *austriacum*	Leaves, stems and twigs	Ethanol 80%	Room temperature	April, June	[84]
*Viscum album*	subsp*. album*	Leaves, stems and twigs	Methanol	High temperature maceration	April, June	[85]
*Viscum album*	n.d.	n.d.	Ethanol	Maceration	n.d.	[86]
*Viscum album*	n.d.	n.d.	Methanol	Household blender	April	[87]
*Viscum album*	n.d.	n.d.	Methanol	Soxhlet extraction	January	[88]
*Viscum album*	n.d.	Leaves	Methanol 70%	High temperature maceration	June	[89]
*Viscum album*	subsp. *album*, *austriacum*, *abietis*	Whole plant	Ethanol 80%	Room temperature maceration	July	[90]
*Viscum album*	n.d.	n.d.	Methanol	Ultrasonic bath (35 °C)	May	[91]
*Viscum album*	subsp. *austriacum*	Leaves and stems	Ethanol	Stirred for 72 h (25 °C)	n.d.	[92]
*Viscum album*	n.d.	Leaves, stems and berries	Methanol 80%, Ethanol 80%	Incubatory rotatory shaker (200 rpm)	December	[93]
*Viscum album*	n.d.	Leaves	Ethanol	Reflux	n.d.	[94]
*Viscum album*	n.d.	Root	Ethanol	Maceration	n.d.	[95]
*Viscum album*	n.d.	Aerial parts	Ethanol	Sonication	February, December	[96]
*Viscum album*	n.d.	Leaves	Ethanol	Soxhlet and ultrasound-assisted extraction	September	[97]
*Viscum album*	n.d.	Whole plant	Ethanol, methanol	Soxhlet	n.d.	[98]
*Viscum album*	subsp. *album*, *austriacum*, *abietis*	Leaves and stems	Methanol 80%	Accelerated solvent extractions	February 2016 to April 2017	[99]
*Viscum album*	n.d.	Leaves and stems	Methanol 85%	Maceration	n.d.	[100]
*Viscum album*	n.d.	Leaves	Methanol	Maceration	April	[101]
*Viscum album*	n.d.	Leaves, fruits and seeds	Methanol	Ultra-Turrax and ultrasonic bath	December	[102]
*Viscum album*	n.d.	Leaves	Methanol	Shaking incubator	n.d.	[103]
*Viscum album*	n.d.	n.d.	Methanol	Soxhlet extraction	January	[104]
*Viscum angulatum*	n.d.	Whole plant	Methanol	Cold maceration	November	[105]
*Viscum angulatum*	n.d.	n.d.	Ethanol, methanol	Cold shaking	n.d.	[106]
*Viscum articulatum*	n.d.	Whole plant	Ethanol	Continuous hot percolation	July, August	[107]
*Viscum articulatum*	n.d.	Aerial part	Methanol 95%	Successive extraction at room temperature	December, July	[108]
*Viscum articulatum*	n.d.	Whole plant	Ethanol 70%	Maceration	February	[109]
*Viscum articulatum*	n.d.	Whole plant	Ethanol	Soxhlet extraction	n.d.	[110]
*Viscum articultum*	n.d.	n.d.	Ethanol	Soxhlet extraction	n.d.	[111]
*Viscum articulatum*	n.d.	Whole plant	Methanol	Maceration at room temperature	n.d.	[112]
*Viscum articulatum*	n.d.	Whole plant	Methanol	Maceration at room temperature	October	[113]
*Viscum articulatum*	n.d.	Aerial parts	Methanol 95%	Maceration at room temperature	December	[114]
*Viscum capense*	n.d.	Stems	Methanol	Soxhlet extraction	n.d.	[115]
*Viscum capitellatum*	n.d.	Aerial parts	80% ethanol, methanol	Decoction	September	[116]
*Viscum capitellatum*	n.d.	n.d.	Methanol	Cold maceration	n.d.	[117]
*Viscum coloratum*	n.d.	Leaves and twigs	Ethanol 75%	Boiling	n.d.	[118]
*Viscum coloratum*	n.d.	n.d.	Ethanol 70%	Maceration at high temperature	n.d.	[119]
*Viscum coloratum*	n.d.	Leaves and twigs	Butanol	n.d.	n.d.	[120]
*Viscum coloratum*	n.d.	n.d.	Methanol 50%	Heated water bath	n.d.	[121]
*Viscum coloratum*	n.d.	Whole plant	Ethanol 95%	Reflux	n.d.	[122]
*Viscum coloratum*	n.d.	Whole plant	Ethanol 50%	Sonication	n.d.	[123]
*Viscum coloratum*	n.d.	Leaves and stems	Methanol	Ultrasonic extraction	n.d.	[124]
*Viscum combreticola*	n.d.	Whole plant	Methanol	Incubatory rotatory shaker (70 rpm)	n.d.	[125]
*Viscum congolensis*	n.d.	n.d.	Ethanol	n.d.	n.d.	[126]
*Viscum cruciatum*	n.d.	Aerial parts	Methanol	Soxhlet extraction	February	[127]
*Viscum cruciatum*	n.d.	Aerial parts	Methanol	Soxhlet extraction	January	[128]
*Viscum cruciatum*	n.d.	Aerial parts	Ethanol 80%	Maceration	April	[129]
*Viscum cruciatum*	n.d.	n.d.	Methanol 80%	Shaking water bath	n.d.	[130]
*Viscum cruciatum*	n.d.	Aerial parts	Methanol	Maceration	n.d.	[23]
*Viscum liquidambaricola*	n.d.	Leaves and stems	Methanol	Ultrasonic extraction	n.d.	[131]
*Viscum monoicum*	n.d.	Whole plant	Ethanol	Maceration	October	[132]
*Viscum multinerve*	n.d.	Leaves and stems	Ethanol	Successive extraction at water bath	n.d.	[133]
*Viscum orientale*	n.d.	Aerial parts	Methanol	Maceration	July	[134]
*Viscum orientale*	n.d.	Leaves	Methanol 100%	Maceration	July	[135]
*Viscum rotundifolium*	n.d.	Leaves	Methanol	Shaking incubator	n.d.	[136]
*Viscum schimperi*	n.d.	Aerial parts	Methanol	Ultra-Turrax	May	[137]
*Viscum schimperi*	n.d.	Aerial parts	Methanol 100%	Ultra-Turrax	May	[138]
*Viscum schimperi*	n.d.	Aerial parts	Methanol	Ultra-Turrax	March	[139]
*Viscum triflorum*	n.d.	Leaves	Ethanol 96%	Ultrasonic bath	February	[140]

### 2.1. In Vitro Studies

#### 2.1.1. Antimicrobial and Antiviral Activities of *Viscum* sp. Alcoholic Extracts

Disc diffusion, agar well diffusion, broth macrodilution and microdilution assays were the major methods employed for the antimicrobial and antiviral activities of *Viscum* sp. alcoholic extracts. From the 14 studies included, 9 were performed with *Viscum album* species. The results revealed a promising antibacterial activity of this plant species, mainly using ethanolic and methanolic *V. album* extracts obtained by maceration and Soxhlet apparatus. A wide variety of bacteria, fungi and yeasts strains were tested: *Aspergillus flavus*, *Bordetella bronchisiptica*, *Bacillus subtilis*, *Candida albicans*, *Escherichia coli*, *Enterococcus faecium*, *Klebsiella pneumoniae*, *Pseudomonas aeruginosa*, *Pseudomonas syringae*, *Staphylococcus aureus*, *Saccharomyces cerevisiae*, *Salmonella typhi*, *Aspergillus niger*, *Agrobacterium tumefaciens*, *Bacillus atrophaeus*, *Bacillus cereus*, *Candida guilliermondii*, *Cryptococcus neoformans*, *Erwinia carotovora*, *Enterobacter cloacae*, *Microsporum canis*, *Proteus mirabilis*, *Staphylococcus epidermis*, *Shigella dysenteriae*, *Salmonella paratyphi* and *Tricophyton mentagropytes*. Hussain et al. showed that the antibacterial potential differed by the part of the plant used to the extract preparation with inhibition zones (IZ) of 15–20 mm to leaves and of 9–24 mm to branches [12]. However, the authors observed that *Aspergillus flavus* and *Saccharomyces cerevisae* were not sensitive to ethanolic and methanolic macerated extracts of the leaves and twigs from *V. album* [12]. Shah et al. also demonstrated the same dependency, with IZ of 15–35 mm to stems, 15–30 mm to leaves and 15–35 mm fruits, according to the microorganism [30]. In this sense, another study using a percolated ethanolic extract of the leaves and stems from *V. album* verified that the activity against the microorganisms was host tree dependent with minimum inhibitory dilution between 0.04 and 3.13% in agar dilution method [26]. Additionally, the antibacterial potential of *V. coloratum* ethanolic extract produced with leaves and stems was tested as a preservative agent in uncooked pork patties during refrigerated storage, which decreased the pH and improved the storage period when compared to the control by inhibition of aerobic microbial multiplication [31]. In contrast, a study using a macerated extract of the *V. album* leaves on quality properties of rainbow trout fillets showed no extended shelf life when compared to control. In this case, both reached up to 20 days [28] (Table 2). The antiviral potency of the 50% hydroalcoholic extract of the leaves from *V. album* subsp. *album* growing on lime trees against parainfluenza virus type 2 in Vero cells was evaluated by Karagöz et al. [32]. The results showed the growth rate and cell viability, in which both were unaffected by the ethanolic extract, and, therefore, it cannot be considered antiviral (Table 2). However, the same study showed that the aqueous extract appeared to have a potent anti-parainfluenza virus potential, demonstrating the influence of the solvent extractor over biological activity. Furthermore, one work demonstrated the antifungal capacity of methanolic extract from *V. album* against *Coniophora puteana*. The authors proved that the use of extract at 18.75% in wood reduces its mass loss promoted by fungi proliferation by approximately 7.97% [103]. In this sense, considering all antimicrobial results included in this review, it was not possible to compare the results obtained by the authors due to the various extraction techniques applied, concentration of the extracts and microorganisms used in the analysis. The majority of the studies evaluated only qualitative aspects through screening tests, such as the disc diffusion assay. They did not present the possible mechanisms of action involved in the biological results and did not present the chemical composition that could be correlated with the observed activity. Additionally, they did not present appropriate positive and negative control groups (Table 2).

#### 2.1.2. Antiparasitic and Insecticide Properties of *Viscum* sp. Alcoholic Extracts

Three studies evaluated the activity of ethanolic extracts obtained by different extraction methods and were tested in different parasites. *V. congolensis* presented anthelmintic activity against earthworms *Alma emini* after 24 h of exposure to the extract, which was compared to albendazole and mebendazole as controls [126]. Despite showing a promising activity, the authors did not demonstrate the parts of the plant used in the study, which could be an obstacle to reproducing the results in future studies. Butanol subfraction extracts prepared with leaves, fruits or berries from *V. album* subsp. *austriacum* (host tree pine) were tested against metronidazole-resistant *Trichomonas vaginalis*. It was observed that 10 mg/mL of butanol subfractions prepared with leaves or berries were more effective in the viability reduction rate of *T. vaginalis* than those prepared with fruits. The authors suggested that the presence of the 2-methylfuran, aromatic heterocycle, in the extracts could be the possible active substance [33]. These findings demonstrate the importance of the plant part description, which is extremely important to the biological activity. Lastly, an ethanol extract prepared with a mixture of leaves, stems and flowers from *V. album* presented 60% of toxicity against *Thaumetopoae solitaria* pupae, which was considered a low insecticide [34] (Table 3). The evaluation of the antiparasitic and insecticide activity of the *Viscum* alcoholic extracts is still incipient, showing a great field of research for the genus *Viscum*.

#### 2.1.3. Cytotoxic and Cytostatic In Vitro Activities of *Viscum* sp. Alcoholic Extracts

The antitumor activity of the mistletoe by its cytotoxicity is dose-dependent [35,36,37,39,40]. Some studies evaluated the influence of the host tree on mistletoe activity. Methanolic extracts from *V. album* berries on *Pinus sylvestris*, *Tilia cordata* and *Populus nigra* presented IC_50_ of 1 mg/mL, 164 µg/mL and 202 µg/mL, respectively, when tested on human colon adenocarcinoma (LS180) by MTT assay [39]. This result demonstrated the influence of the host tree in the *V. album* cytotoxic activity, and the extracts produced by *V. album* from *Pinus sylvestris* presented lower biological activity, probably due to the difference in chemical aspects. In addition, the authors verified through BrdU assay that the extracts’ cytotoxicity did not have correlation with alterations in DNA synthesis [39]. In another study, methanolic extracts from *V. album* leaves growing on *Tilia argentea*, *Acer campestre* subsp. *campestre* and *Robinia pseudoacacia* decreased the viability measured by MTT assay of human cervical carcinoma (HeLa) with IC_50_ of 93 µg/mL, 165 µg/mL and 85 µg/mL, respectively [37]. Holandino et al. showed that ethanolic extract from *V. album* subsp. *album* was more cytotoxic to Molt-4 and Yoshida cancer cell lines than extracts from subspecies *abietis* and *austriacum* [90]. These results confirmed the importance of the host tree description for the biological application. Methanolic extract of the aerial parts of *V. cruciatum* at 30 µg/mL exhibited a moderate degree of growth inhibition on human laryngeal carcinoma cells (HEp-2) after 72 h of the incubation when compared to 6-mercaptopurine positive control [127]. The authors associated the result with the presence of β-Amyrin acetate that presented a greater cytostatic action than the control. Yang et al. showed that isolated terpenes, mainly 3-*epi*-betulinc acid, oleanolic acid and erythrodiol from the *V. coloratum* ethanolic extracts, produced with leaves and twigs, were more potent on human ovarian carcinoma (HO-8910) and human hepatocarcinoma (SMMC-7721), with lower IC_50_ when compared to the full extract [118]. Another isolated compound (hirsutanone) from aerial parts of *V. cruciatum* methanolic extract possessed cytotoxic activity against human melanoma (UACC-62), renal adenocarcinoma (TK-10) and breast adenocarcinoma (MCF-7) cell lines, with IC_50_ values of 4.8, 6.8 and 1.9 µg/mL, respectively [128]. The anticancer activity of the isolated hirsutanone of the *V. cruciatum* was associated with apoptosis induction. *V. album*’s apoptotic induction was identified by reduction of Hsp 27 and 14-3-3 and induction of caspase-3 proteins expression in rat glioma cells (C6) [36] and by increasing the cells in Sub G0 cycle, which affected the cell cycle in an early apoptotic activity in murine melanoma (B16F10) [35]. Only one study demonstrated the necrotic cytotoxicity of the *Viscum album* alcoholic extracts [90]. *V. coloratum* ethanol extracts (100–200 µg/mL) were not cytotoxic to the cell-derived inflammatory mediator (MDIM)-activated cells and human colorectal adenocarcinoma (Caco-2) cells despite having decreased the inflammatory processes in different ways [14,119] (Table 4). Methanolic extract of the *Viscum cruciatum* from almond tree was able to arrest the cell cycle of the MCF-7 (breast adenocarcinoma) in the G0/G1 phase. According to the authors, 15 flavonoids present in this extract inhibited CDK2, CDK4 and CDK6 proteins that are cell cycle checkpoint proteins in the Cyclin/CDK pathway [23].

The majority of studies evaluated the cytotoxic and cytostatic activity in human and rat cell lines by MTT assay. The findings demonstrated that the cells’ response to stimulus depends on the *Viscum* species, as well as the host tree, plant parts used in the extract production and on the concentration of the extract analyzed. Most of the described active compounds are from the flavonoid and phenolic acid classes.

#### 2.1.4. Cell Migration and Metalloproteinases Inhibition Induced by *Viscum* sp. Alcoholic Extracts

Metalloproteases (MMP) are elevated in the cartilage tissues and joint fluids of humans with rheumatoid arthritis, which can be responsible for cell migration and cartilage degradation. In this sense, *V. coloratum* ethanol extract inhibited cell migration of MDIM-stimulated chondrocyte cells (SW1353) and a reduction in secretion and activity of MMP-1, MMP-3 or MMP-13. Thus, these results suggest its anti-osteoarthritic activity [14]. However, in future studies about this important biological activity, it will be necessary to provide information about the parts of the vegetal species used, as well as the plant’s harvest season, in order to ensure the reproducibility of results. In another study, EtOH extract prepared with roots of *Viscum album* inhibited MMP-13 expression in 64.3%, 70.3% and 80.0% at 50, 100 and 200 μg/mL, respectively [95] (Table 5).

#### 2.1.5. Antiplatelet and Antihypertensive Activities of *Viscum* sp. Alcoholic Extracts

The pharmacological activity of phenylpropanoids isolated from leaves and stems of the *V. album* from *Pyrus caucasica* inhibited ADP-induced platelet aggregation in a concentration range from 0.001 to 1.0 µM [21]. Another species, *V. cruciatum*, also inhibited adrenaline and ADP-induced platelet aggregation in a dose-dependent manner by the aerial parts ethanol extract [129]. A dose-dependent inhibition of *V. album* ethanol extracts prepared with leaves and stems on rabbit platelet aggregation was higher to the platelet-activating factor (PAF) compared to the ADP and arachidonic acid (ARA) pathways, significantly decreasing thromboxane A2 (TXA_2_) production [41]. Host tree-dependent activity was evaluated, with *V. album* from olive and almond host trees, in which both presented prolongation of prothrombin time (PT) and activated partial thromboplastin time (aPTT), important indicators of coagulation [25]. Although the first study describes the potential of phenylpropanoids as the main pharmacological compound, the other three did not mention possible chemical classes or compounds responsible for the observed biological activity [25].

In relation to antihypertensive activity by inhibition of the angiotensin-converting enzyme, the *V. triflorum* ethanol extract from different host trees did not present 50% or more of inhibition activity at 0.33 mg crude extract in 1 mL test solution. However, the authors demonstrated that six aqueous samples of *V. triflorum* from *Acacia heterophylla* host tree showed 64–87% of enzyme inhibition [140]. It was possible to observe that the *V. album* has been the most studied species for platelet aggregation and coagulation, showing a lack of studies with the other alcoholic *Viscum* species (Table 6). Despite of the folk description of the alcoholic *Viscum* extracts to reduce blood pressure [141], this review highlights the necessity of more in vitro studies to better understand the mechanism of action involved in the treatment of hypertension by the genus *Viscum* and its extractive solvents.

#### 2.1.6. Anti-Inflammatory Effects Activities of *Viscum* sp. Alcoholic Extracts

Many studies have focused on finding new anti-inflammatory medicinal plants since the known anti-inflammatory drugs promote many side effects. Hence, the anti-inflammatory potential of ethanol and methanol extract produced with the *V. articulatum* whole plant was observed, mainly due to the presence of flavonoids and triterpenoids [109]. *V. coloratum* ethanol extract reduced the inflammatory responses in inflammatory bowel disease (IBD) by suppressing MMP-2 and MMP-9 expression in Caco-2 cells and recovering the expression of zonula occludens-1, a tight junction protein. These results were associated to the high content of flavonoids [119]. Another study with *V. coloratum* ethanol extract observed strong inhibitory action in ß-hexosaminidase activity and TNF-α, IL-4, PGD2 and LTC4 formation, blocking the expression of COX-2 and the phosphorylation of 5-lypoxigenase, spleen tyrosine kinase, PLCgama1, PKCδ, Akt, JNK, ERK and p38, thus exerting anti-allergic and anti-osteoarthritic actions [14]. In contrast, ethanolic extracts from *V. album* subsp. *album*, subsp. *abietis* and subsp. *austriacum* exhibited almost no remarkable inhibitory activity on the inflammatory cytokines (IL-1α, IL-1β, TNF-α) at tested concentrations [43] (Table 7). *V. album*, *V. articulatum* and *V. coloratum* were the species better studied for the anti-inflammatory properties. However, considering the six studies included, only three described the parts of the plant used that were very different from each other (leaf, flower and whole plant). In this sense, the results present an interesting pharmacological potential of these species as an anti-inflammatory remedy, but further studies with more details are necessary.

#### 2.1.7. Immunomodulatory Effects of *Viscum* sp. Alcoholic Extracts

Peripheral blood mononuclear cells and neutrophils are crucial immune cells that carry out host defense. Human CD69 is an important antigen responsible for T-cells activation. In this sense, an immunomodulatory effect of the *V. album* subsp. *album* methanolic extract (1 mg/mL) was observed by increased CD69 expression in peripheral mononuclear blood cells, which mediates the activation of CD4^+^, CD25^+^, CD8^+^ and CD25^+^ T cells. The extract also increased the phagocytosis of *Candida albicans* blastospores and intracellular killing function of neutrophils compared to negative control [44]. These extracts can be considered as a good option for new investigations working on immune system activation, but it is necessary to standardize the host tree and the parts of the plant used, as well as include positive controls for better comparison. Moreover, the authors did not present a chemical composition to correlate with the observed activity.

#### 2.1.8. Hypoglycemic/Hypolipidemic Activities of *Viscum* sp. Alcoholic Extracts

*V. schimperi* decreased advanced glycation end products [137], and *V. album* rich in phenolic compounds showed a potent anti-glycation activity [45]. *V. album* subsp. *album* presented low inhibitory potential on α-amylase and on α-glucosidase activities and thus might ameliorate hyperglycemia in diabetes type 2 [46]. In complement, *V. articulatum* ethanolic extract was considered moderately suitable for controlling diabetic conditions by its inhibitory activity on α-amylase [110]. An anti-obesity application of *V. album* was observed by its inhibitory effect on pancreatic lipase [47] (Table 8).

#### 2.1.9. Cellular Antioxidant Effect of *Viscum* sp. Alcoholic Extracts

Oxidative stress causes damages that trigger many disorders. *V. coloratum* ethanol extract presented an inhibitory effect on tyrosinase and Superoxide dismutase (SOD)-like stimulation activity [49]. Important enzymes involved in the oxidative stress, SOD-like, catalase (CAT) and glutathione reductase (GR) activities decreased significantly in two different studies [13,104]. Chromosomal aberrations and mitotic index were dose-dependent, and malondialdehyde decreased with *V. album* extract, suggesting the extract’s antioxidant, anti-mutagenic and DNA-repairing mechanism-inducing properties [42]. *V. album* methanolic extracts did not affect the steady-state level of intracellular ROS, but the activity was host tree-dependent. Pre-treatment with *V. album* from *Robinia pseudoacacia* and *Tilia argentea* completely prevented the damage on nuclear and mitochondrial DNA under stress conditions, decreasing ROS formation, while the one from *Acer campestre* host tree was effective against nuclear DNA but partially for mitochondrial DNA damage [37]. Lastly, pork meat quality was tested by using *V. album* extract, which was highly effective in maintaining uncooked pork patties by inhibiting lipid oxidation and preventing odor development, which was higher than the control ascorbic acid [31] (Table 9). All studies included in this section evaluated the *V. album* species, showing the necessity to research the other species, as well as different parts of the plant and season harvest.

### 2.2. In Vivo Studies

#### 2.2.1. Hypoglicemic Effects of *Viscum* sp. Alcoholic Extracts

Methanol extracts of the aerial parts of *V. schimperi* and *V. album* and ethanol extract of the leaves of *V. album* reduced the glucose level in a dose and/or time-dependent manner [50,51,138,139]. Furthermore, *V. colaratum* enhanced insulin secretion 0.82 ± 0.14 ng/mL (control) to 1.07 ± 0.19 ng/mL (ethanolic extract) in partial pancreatectomized rats probably by the increase in β-cell proliferation [52,138]. The effects in glucose level reduction were also dependent on the *Viscum album* subsp. and host trees. Extracts reduced the blood glucose level in streptozotocin-induced diabetics rats from 329.8 mg/dL (control) to 289 mg/dL, 286 mg/dL and 243 mg/dL to *V. album* subsp. *abietis*, *album* and *austriacm*, respectively [53] (Table 10).

#### 2.2.2. Hypolipemic Effects of *Viscum* sp. Alcoholic Extracts

*V. album* subsp. *album* was able to reduce serum cholesterol, LDL-C and triglyceride more than 50% and increase serum HDL-C in 46.7% in male Swiss albino mice. *V. schimperi* methanolic extract also decreased total cholesterol, LDL-C and triglyceride around 30% and elevated the HDL-C to 171.5% [54,139]. However, Vadnere et al. did not observe hypolipemic activity of ethanol extract from *V. articulatum* [111] (Table 11). These results demonstrated that *V. album* and *V. schimperi* could be good sources with hypolipemic action.

#### 2.2.3. Anticancer Activities of *Viscum* sp. Alcoholic Extracts

The number of studies related to the in vivo activity of aqueous *Viscum* extracts is enormous. Bonamin et al. in a recent review showed that different aqueous preparations of the *Viscum* were able to reduce tumor growth and tumor cell viability, increase tumor necrosis and also promote tumor angiogenesis reduction [142]. However, in relation to alcoholic extract, few articles were found to highlight the necessity of more studies (Table 12). In the present review an association of *V. album* ethanol extract with doxorubicin was able to reduce Ehrlich ascites carcinoma volume and catalase and xanthine oxidase activity [55,56].

#### 2.2.4. Hypotensive Effects of *Viscum* sp. Alcoholic Extracts

Methanol extract of *V. angulatum* and *V. articulatum* had a significant dose-dependent effect on the urine excretion volume with diuretic index of 2.76 and 3.00, respectively, in relation to control. An increased natriuretic (Na^+^/K^+^) and saluretic (Na^+^ + Cl^−^) index were observed for both species, but the *V. articulatum* extract presented a higher saluretic index of 272 when compared to 168 of the *V. angulatum* extract [105,112]. Supporting this data, Bachhav et al. 2012 also demonstrated that the methanol extract of *V. articulatum* prevented the progression of hypertension in rats due to urine volume and Na^+^ rising [113]. In addition, *V. album* ethanol extracts presented a hypotensive dose-dependent response in rats with maximal reduction at 1 × 10^−3^ mg/kg/b.w. The blood pressure reduction was 23.56 mmHg with effect via muscarinic receptors [15] (Table 13). In this sense, *V. angulatun*, *V. articulatum* and *V. album* are the main species on which studies for antihypertensive activity and have promising data that support future clinical trials.

#### 2.2.5. Analgesic and Anti-Inflammatory Activities of *Viscum* sp. Alcoholic Extracts

*V. monoicum* ethanol extract showed a potential increase in pain threshold analgesia by tail immersion test in a dose-dependent manner of the 5.05 ± 0.43 and 5.81 ± 0.23 seconds at 200 and 400 mg/kg/b.w., respectively, as compared to pentazocine (6.29 ± 0.28 seconds) at 10 mg/kg/b.w. (*p* > 0.05) after 120 minutes [132] (Table 3). *V. album* methanol extract of the whole plant exhibited significant analgesic activity in tail immersion test with respect to the control group, but none of the doses showed effects comparable to the standard drug [57]. Methanol extract of *V. orientale* at 500 mg/kg/b.w. inhibited 88.8% of the acetic acid induced writhing, demonstrating analgesia by prostaglandin synthesis inhibition. At the same dose, the extract inhibited paw licking in the formalin induced pain model 56.4% in the early phase and 72.6% in the late phase, respectively [135].

Lin et al. 1994 [133] demonstrated that ethanol extract of *V. multinerve* (100 and 300 mg/kg/b.w.) promoted anti-inflammatory activity by reduction of carrageenan-induced edema compared to indomethacin. However, the extract was not effective in protecting the liver against CCI_4_-induced damage. *V. coloratum* ethanol extract demonstrated activity in inflammatory bowel disease since it significantly attenuated enterorrhagia and colonic edema in Dextran Sodium Sulfate (DSS)-induced colitis. This activity was produced in mice by inhibiting the immune cells infiltration in IBD and by decreasing the levels of immunoglobulin E, IL-6 and TNF-*α* [119] (Table 14).

#### 2.2.6. Neuropharmacological Activities of *Viscum* sp. Alcoholic Extracts

*V. capense* methanol extract significantly delayed the onset of pentylenetetrazole- and bicuculline-induced tonic seizures. At 100 mg/kg/b.w. intraperitoneal (i.p.) it significantly attenuated the seizures reducing the number of animals convulsing. However, the same extract did not alter N-methyl-*DL*-aspartic acid-induced tonic seizures [115]. *V. album* methanol extract reduced rearing and crossings with respect to control in open field test, suggesting central nervous system (CNS) depressant activity [57]. Khatun et al. 2016 [135] also showed that the *V. orientale* methanol extract exhibited CNS depressant activity by decreasing exploratory behavior in mice, as well as the frequency and amplitude of the movements.

Genus *Viscum* alcoholic extracts were also able to reduce anxiety, depression and stress in different animal models. *V. album* methanol extract at 100 mg/kg/b.w. reduced the anxiety in mice by the increased number of entries and time spent in open arms of elevated plus maze (EPM), statistically equivalent to the standard drug. The authors also showed antidepressant activity using despair swim test after acute administration of methanol extract (200 or 400 mg/kg/b.w., p.o.), which significantly reduced the mice immobility time at all doses with respect to control. However, none of the doses showed activity equivalent to the standard drug. Moreover, the methanol extract significantly reduced the time spent by mice in an immobile state in cold swim test with respect to control, showing a mild anti-stress activity that was not equivalent to the standard drug [57]. At higher doses, the methanol extract was able to promote significant hypnotic activity by increasing the duration of sleep in mice in thiopentone sodium-induced sleeping assay [57] (Table 15).

#### 2.2.7. Toxicity Activities of *Viscum* sp. Alcoholic Extracts

*V. coloratum* crude ethanol extract promoted mice mortality in acute toxicity test by using intragastric administration (LD_50_ 7.67 g/kg/b.w). However, with the action of the *Rhodobacter sphaeroides* in the extract, for a fermentation process, it reduced the number of deaths to zero [118]. This result suggests that the chemical composition was changed by fermentation reducing the toxicity of the extract, probably by biotransformation of toxic proteins. Methanol extract of the *V. album* subsp. *album* at 250 mg/kg/day showed protective effects against cyclophosphamide-induced cardiotoxicity, urotoxicity and genotoxicity in mice. The authors demonstrated that the extract improved the levels of antioxidant enzymes (superoxide dismutase, catalase and glutathione peroxidase, Glutathione-S-transferases, reduced glutathione) and mitotic activity of bone marrow cells. Furthermore, the pre-treatment with *V. album* together with cyclophosphamide significantly decreased aberrant cells and chromosome aberrations when compared to cyclophosphamide alone [58]. *V. album* methanolic extract at 250 mg/kg/b.w. had a protective effect against methotrexate-induced cyto-genotoxicity in mouse bone marrow, decreasing the number of chromosomal aberrations of 96.40 ± 8.25 to 59.20 ± 1.65 in relation to methotrexate. The extract also significantly increased the mitotic index from 33.12 ± 1.79 to 60.12 ± 1.12 in bone-marrow cells that had been suppressed by methotrexate compared to a positive control [59] (Table 16).

#### 2.2.8. Other Activities of *Viscum* sp. Alcoholic Extracts

Methanol extract of *V. coloratum* at 125, 250 and 500 mg/kg/b.w. prolonged the bleeding time in transected rat tail, suggesting its use in improving blood circulation [60].

A flavanone (homoeriodictyol-7-*O*-β-D-glucopyranoside) isolated from *n*-butanol extract of *V. coloratum* was pharmacokinetically observed in rat plasm after intravenous administration. The compound presented T1/2,α and t1/2,β of the 0.06 ± 0.01 h and 1.27 ± 0.31 h, and was mainly distributed to the liver and small intestine [120].

Karagöz et al. [61] showed that methanol extract of *V. album* subsp. *album* improved parameters of heart failure in rats, such as left ventricular diameters, ejection fraction, serum NT-proBNP (N-terminal pro b-type natriuretic peptide) levels and histopathological changes. This resulted in a statistically significant attenuation of increased levels of nitric oxide 48.5 ± 2.3 μmol/L to isoproterenol group when compared to the control (19.0 ± 3.3 μmol/L) and *V. album* group (37.8 ± 3.1 μmol/L). The levels of high-sensitivity C-reactive protein were also found to be lower in the *V. album* group (0.133 ± 0.023 ng/mL) when compared to the control (0.183 ± 0.034 ng/mL) and isoproterenol group (0.155 ± 0.025 ng/mL) (Table 17).

### 2.3. Ex Vivo Studies with Viscum sp. Alcoholic Extracts

Six studies evaluated the ex vivo vascular, antispasmodic and antidiabetic activities. Ethanol and *n*-butanol extracts of *V. album* promoted contraction in noradrenaline-contracted rat aortic rings that were host tree- and dose-dependent [62]. Corroborating with this data, Deliorman et al. also showed that polar subfractions of *V. album* subsp. *album* ethanol extract and some isolated compounds (Syringin, Coniferin, 5,7-dimethoxy-flavanone-4′-*O*-[β-D-apiofuranosyl-(12)]-β-D-glucopyranoside) produced contractile responses in a dose-dependent manner in noradrenaline-contracted rat aortic rings [63]. In addition, the vasodilator activity was observed in the less polar subfractions. However, the Kalopanaxin D (hydroxycinnamic acid derivative) displayed a very slight relaxant response. In this context, *V. album* methanol extract partially inhibited phenylephrine (1 μM) and K^+^ (80 mM)-induced sustained contractions of the rabbit aortic ring through the blockade of Ca^++^. Additionally, considering the action in cardiovascular diseases, Suveren et al. showed that methanolic *V. album* extract at 5 mg/L mediated the nitric oxide-dependent cardioprotection against myocardial injury originated by ischemia/reperfusion insult, reducing 53.2% in mean infarct size compared to control hearts [64]. Regarding antispasmodic activity, *V. album* methanol extract relaxed the spontaneous and the K^+^ (80 mM)-induced sustained contractions of rabbit jejunum in a dose-dependent manner with an EC_50_ value of 0.31 mg/mL (0.15–0.57) and 0.62 mg/mL (0.3–0.95), respectively, through the blockade of Ca^2+^ [65] (Table 18).

Similarly, Gilani and coworkers also found a concentration-dependent (0.01–3.0 mg/mL) relaxation of spontaneous and K^+^ (80 mM)-induced contractions of isolated rabbit jejunum by the *V. cruciatum* ethanol extract with action in Ca^2+^ channels [129]. Additionally, a spasmogenic effect was observed with a concentration-dependent contractile effect in guinea-pig ileum at 5–10 mg/mL of the extract [129] (Table 18).

### 2.4. Clinical Trial

One clinical pilot study of *V. album* mother tincture was evaluated on 41 newly diagnosed hypertensive patients who had not taken any medication [66]. Blood pressure was taken for the following 3 weeks, after which the treatment started and lasted for 12 consecutive weeks (10 drops in 30 mL of water 3 times a day, half an hour after food). Fifty-nine percent of patients were at stage 1 and 33% at stage 2 hypertension, after which the systolic blood pressure decreased from 155.8 mm Hg to 141.5 mm Hg, as well as diastolic pressure from 84.4 mm Hg to 79.5 mm Hg (*p* < 0.05). Serum cholesterol was not altered, but a reduction in serum triglyceride was significant (*p* < 0.001). Serum lactate dehydrogenase and serum urea significantly rose, however not higher than the normal level. None of the patients presented any sign of cardiac or musculoskeletal discomforts. Considering these results, *V. album* mother tincture could be used to regulate blood pressure and to lower serum triglyceride levels [66]. However, this study did not consider the *Viscum album* subspecies and its host tree. Furthermore, the authors chose a 1-group pretest–posttest study and did not include a control group and a blinding method. Thus, more clinical studies are necessary, in addition to better methodological design.

### 2.5. Chemical Aspects of Viscum sp. Alcoholic Extracts

The chemical composition of natural products is an essential step in the evaluation of their biological potentialities. Studies focused on phytochemical profile and antioxidant activities of the vegetal extracts have been published over the years. *V. album* has received remarkable attention due to its effectiveness in clinical oncological therapy [143]. The present review showed different chemical classes present in *Viscum* sp. with antioxidant properties: flavonoids (e.g., flavanones and flavonols), phenolic acids and triterpenes. In addition, the total content of chemical groups such as total pro-anthocyanidin content (TPAC), total flavonoid content (TFC), total carotenoid content (TCC), total triterpene content (TTC) and experiments applied to antioxidant capacity assay in alcoholic extracts of *Viscum* species are shown in Table S1.

The total phenolic content (TPC) was expressed mainly in gallic acid with some exception in caffeic acid, *p*-coumaric acid, quercetin, tannic acid or catechin/dry weight or fresh weight. Others, such as the total flavonoid content (TFC), were expressed mainly by quercetin content but also used rutin and kaempferol/dry weight of plant. Following, total pro-anthocyanidin content (TPAC), total carotenoids content (TCC) and total terpenoid content (TTC) used catechin, β-carotene and oleanolic acid, respectively.

Upon the evaluation of these results in relation to the different total contents in each chemical group by species, the quantitative results varied considerably among authors, especially in the *V. album*, which may be due to the type of cultivation, geographical origin, host tree, climatic conditions and different extraction procedures [3]. The total phenolic content in this species ranged from 0.19 [71] to 1232 [68] mg/g dry extract or fresh weight. Stefanucci and coworkers showed that extracts from *Viscum album* leaves had more phenolic and flavonoid content than fruits and seeds. The authors also highlighted that the method of extraction promoted differences in these contents [102]. Furthermore, the other species of *Viscum* also demonstrated the same pattern, with great variability in the phenolic composition. This characteristic can be explained by differences in the site harvest, host tree and parts used of the plant.

Considering that the total content experiments are the preliminary assay to guide authors in antioxidant evaluation, in vitro colorimetric methods were carried out. Some studies used a unique antioxidant assay model that was not conclusive [130,135,137]. Nevertheless, in the case of *V. album,* 23 different in vitro assay models have been performed, all of them detailed in Table S1. It is difficult to fully compare one method to another because some differences in the reaction mode, procedure, sample, chemical reagent, etc. [144,145]. The in vitro colorimetric methods that were most frequently used and were put in order of decreasing frequency are described as follows: Folin–Ciocalteu reducing capacity assay; DPPH; ABTS^•+^ (radical cation 2,2-azinobis-(3-ethylbenzothiazoline-6-sulphonic acid) scavenging activity); SRSA (Superoxide anion radical scavenging activity); FRAP (ferric reducing antioxidant power) assay and others such as TEAC (Trolox Equivalent Antioxidant Capacity); β-Carotene-linoleic acid assay; SOD (Superoxide dismutase assay); ORAC (Oxygen Radical Absorbance Capacity) assay; α-amylase/α-glucosidase inhibitory activity; chelation power on (Fe^2+^) ions activity assay; FRSA (Free radical scavenging activity) assay; HRSA (Hydroxyl radical scavenging activity) assay; H_2_O_2_ Hydrogen peroxide scavenging activity assay; DMPD (N,N-dimethyl-p-phenylenediamine radical scavenging activity) assay; NO (Nitric oxide radical scavenging activity) assay; PRAP (Phosphomolibdenum-reducing antioxidant power) assay; AChE, BChE, and Tyrosinase activity assays; TBARS (Thiobarbituric acid reactive species) assay; XOD (Xanthine oxidase) assay; Chemiluminescence; FTM Ferric thiocyanate method (Table S1). *V. album* was widely studied in in vitro antioxidant assay in comparison to the other species, and DPPH assay was the most common method applied. In addition, considering the DPPH, for the same *Viscum* sp. some authors used the IC_50_ value [68,70,74] to represent the antioxidant capacity, but they used different equivalent of a specific antioxidant standard, such as rutin, quercetin, chlorogenic acid, etc [46,49,91]. This review shows that a standardization of the antioxidant methods, in which their results can be comparable to each other, is very important. Finally, as described above, the antioxidant activity can be obtained by multiple ways using a variety of experimental procedures, making it difficult to compare these experimental data.

Some studies performed only preliminary phytochemical analysis of alcoholic extracts in six species of the genus *Viscum*: *V. album* [30,50,79], *V. articulatum* [107,110], *V. monoicum* [132], *V. congolensis* [126], *V. cruciatum* [129], *V. orientale* [134]. They revealed the presence of chemical classes such as alkaloids, flavonoids, terpenoids, tannins, glycosides, cardiac glycosides, anthraquinone glycosides, reducing sugar, emodin, coumarin, phenols and proteins (Table S2). Sometimes, the authors associated this preliminary chemical composition with the observed biological activities. Additionally, many articles deepened their chemical studies of the isolation and characterization of the bioactive compounds in *Viscum* species. In this sense, the studies have isolated many compounds from different parts or the whole plant. The identification of some primary metabolites, such as fatty acids (n = 11) [80,138], carbohydrates (n = 7) [81], and other miscellaneous (n = 8) has been reported [33,75,108,128]. Nevertheless, secondary metabolites, such as flavonoids (n = 70) [45,73,78,138], phenolic acids (n = 35) [35,74,116] and terpenoids (n = 16) [82,114,127] were the ones mostly identified (Figure 4A). These are compiled in the Supplementary Material (Tables S3–S8). Moreover, these compounds are summarized in Figure 4B, where the *V. album* was predominant studied followed by *V. schimperi* and *V. coloratum.*

It was observed that flavonoids were the most commonly identified substances in alcoholic extracts from *Viscum* species [35,45,73,75,78,138]. Phytochemical studies in *Viscum* genus have reported the predominance of subclasses such as flavanone, flavonol, flavone and their respective glycosylated derivatives (Table S6). In addition to flavonoids, a series of phenolic compounds, mainly phenylpropionic and benzoic acids and their glycosylated derivatives, have been detected. Syringenin, syringenin-apiosylglucoside and some lignans, such as eleutheroside E and syringaresinol monoglucoside, were identified in alcoholic mistletoe preparations [21,35,80,82,84,85] (Table S5). Our review shows that flavonoids were the largest group of secondary metabolites, followed by phenolic acids and terpenoids (Figure 4), which provided target pharmacological profiles such as antioxidant, antihypertensive, antidiabetic, anti-inflammatory and others. Regarding the solvent’s physicochemical properties, these were vital for the extraction of these chemical groups. Alcohols (EtOH and MeOH) are polar solvents extensively used to extract antioxidant compounds due to their effectivity in extracting phenolic compounds [19,20].

A great number of studies identified terpenoids in alcoholic extracts (Figure 4) (Table S7). For instance, oleanolic acid was observed in *V. album*, *V. angulatum*, *V. articulatum*, *V. capitellatum*, *V. coloratum*, *V. schimperi* [52,80,82,112,113,114], and betulinic acid in *V. album*, *V. angulatum*, *V. articulatum*, *V. capitellatum*, *V. coloratum*, *V. schimperi* [80,83,105,112,113,116,118,137,138].

Moreover, fatty acids were identified in *V. album* and *V. schimperi* (Table S3) and carbohydrates in *V. album* extracts (Table S4), the main studied species of Viscum. In addition, Čiča et al. identified a total of 166 volatile metabolites in commercial and homemade preparations, alcoholic beverages called “*Biska*”, produced from *V. album* (esters, alcohols, terpenes, aldehydes, ketones, alkanes, acetals, acids) using GC-MS [86] (Table S8). Other volatile compounds, such as naphthoquinone (*V. album*), methoxyphenol (*V. articulatum*), unsaturated organic acids (*V. album*) and one diarylheptanoid (*V. cruciatum*) were also reported [75,108,128] (Table S8).

Information about analytical methods is detailed in Tables S3–S8. Instruments such as high-performance thin-layer chromatography (HPTLC) and high-performance liquid chromatography (HPLC), coupled to ultraviolet (UV), diode array detectors (DAD) or mass spectrometry (MS), have contributed to the characterization of phenolic acids and flavonoids. In the case of terpenoid compounds, nuclear magnetic resonance spectroscopy (1D and 2D NMR) and gas chromatography (GC) were the most used techniques. Additionally, primary metabolites such as fatty acids and carbohydrates were identified with the aid of instruments, such as LC-MS, NMR and GC-MS. In the last decades, a dramatic acceleration in the development of new technology of separation, detection and preparation of biological samples was achieved, which brought as a benefit the characterization of hundreds of phytochemical constituents in a single analysis. In this sense, this review showed that most of the chemical compounds had been isolated mainly from *V. album*, suggesting that other studies regarding chemical characterization from other species need to be explored. 

## 3. Material and Methods

A literature search was performed using PUBMED, EMBASE and SCOPUS databases up to 31 August 2022. A starting period or language filters were not used. The following search strategy was used: (1) *Viscum* OR mistletoe; (2) alcoholic OR ethanol OR ethanolic OR EtOH OR methanol OR methanolic OR MeOH OR butanol OR butanolic OR BuOH OR tincture OR mother tincture; (3) 1 AND 2. The inclusion criteria considered publications about chemical, in vitro, in vivo, ex vivo and clinical studies for *Viscum* sp. alcoholic extracts. Publications were excluded if the content was about ethnopharmacological studies, reviews, short lectures or abstracts, in addition to studies that presented a mixture of plants or non-alcoholic *Viscum* extracts. Furthermore, studies in languages in which the authors were not proficient (Chinese, Japanese, Korean, Persian or Turkish) and articles that did not provide free access were excluded. First, the titles and abstracts were evaluated by two authors through the Rayyan Systems Inc. tool (https://www.rayyan.ai/, accessed on 6 March 2023). Second, the authors screened the selected ones and excluded non-alcoholic *Viscum* studies. Then, all publications were checked for eligibility. At this point, all relevant publications were checked by two authors independently based on the inclusion criteria. A third author evaluated the publication if disagreements occurred. The main results of included publications were summarized in a new matrix, specified in categories (Table 1, Table 2, Table 3, Table 4 and Tables S1–S8/Supplementary Material).

## 4. Conclusions

This review showed that *Viscum* sp. alcoholic extracts presented positive and promising activity in hypertension, dyslipidemia, inflammation and diabetes, among other health disorders. Fourteen *Viscum* species were identified, and *Viscum album* was the main species studied followed by *Viscum articulatum*, showing a gap in studies with different *Viscum* sp. alcoholic extracts. Flavonoids, phenolic acids and terpenoids were the most described chemical classes in these species, with great potential for biological applications, such as antioxidant, antimicrobial and hypolipidemic. Only one clinical study with alcoholic extract of the *Viscum album* was found, which is in opposition to hundreds of clinical studies with aqueous extracts. This big gap needs more attention and research to support the folk use of these ethanolic extracts, especially as a homeopathic remedy. Some studies did not present important aspects of the raw material, such as season and site of harvest, subspecies and detailed extractive method, which is crucial for their pharmacological uses, reducing the quality of the studies and making their reproducibility difficult. Thus, our study contributes to the genus *Viscum* literature by compiling the state of the art and can be used as a database for planning new research in the scope of alcoholic extracts from *Viscum* species.

## Figures and Tables

**Table 2:** *Viscum* sp. in vitro activities—Antimicrobial and antiviral.

Species	Assay	Microorganism/Virus	Main Results	Authors
*V. album*	Disc diffusion assay	*Alcaligenes feacalis*, *Acinetobacter lwoffi*, *Bacillus cereus*, *Bacillus subtilis*, *Cladosporium herbarum*, *Escherichia coli*, *Klebsiella pneumonia* subsp*. pneumonia*, *Pseudomonas aeruginosa*, *Providencia alcaliaciens*, *Penicillium roquefortii*, *Proteus vulgaris*, *Staphylococcus hominis*	MeOH extract had activity against 12 out of 32 microorganisms tested (IZ 1–11 mm).	[29]
*V. album*	Colony formation assay	n.d	CFU changed during the storage period of the 14 days, and it was lower than the positive control. Log CFU in 14 days: positive control 5.27 ± 0.41 × 5.06 ± 0.18 of the extract 1%.	[31]
*V. album*	Disc diffusion assay	*Aspergillus flavus*, *Bacillus subtilis*, *Bordetella bronchisiptica*, *Enterococcus faecium*, *Escherichia coli*, *Salmonella typhi*, *Pseudomonas aeruginosa*, *Pseudomonas syringae*, *Saccharomyces cerevisae*, *Staphylococcus aureus*	EtOH and MeOH extracts (100 mg/mL) presented different inhibitory activity according to plant parts used: leaves IZ 15–20 mm, branches IZ 9–24 mm. *Saccharomyces cerevisiae* and *Aspergillus flavus* were not sensitive to extracts in the tested conditions.	[12]
*V. album*	Agar dilution method	*Candida guilliermondii*, *Cryptococcus neoformans*, *Microsporum canis*, *Tricophyton mentagropytes*	EtOH extracts were potent against all tested microorganisms with minimum inhibitory dilution between 0.04 and 3.13%. Activity was host tree dependent.	[26]
*V. album*	Disc diffusion assay	*Agrobacterium tumefaciens*, *Bacillus atrophaeus*, *Bacillus subtilis*, *Candida albicans*, *Erwinia carotovora*, *Escherichia coli*, *Klebsiella pneumoniae*, *Pseudomonas aeruginosa*, *Salmonella typhi*, *Staphylococcus aureus*	MeOH and BuOH extracts showed activity against all Gram-positive and Gram-negative bacteria and fungus tested and were dependent of the plant part used and the microorganism evaluated: stems IZ 15–35 mm; leaves IZ 15–30 mm; fruits IZ 15–35 mm.	[30]
*V. album*	Agar dilution method	Lactic acid bacteria, *Enterobacteriaceae*	Latic acid bacteria counts in negative control and *V. album* group were 2.67 and 2.44 log CFU g^−1^ at 6 days in the storage period. *Enterobacteriaceae* count in control and *V. album* group reached 7.05 and 7.54 log CFU g^−1^ at 23 days, respectively.	[28]
*V. album*	Disc diffusion assay and MIC in 24 well plate	*Aspergillus niger*, *Bacillus subtilis*, *Candida albicans*, *Esherichia coli*, *Pseudomonas aeruginosa*, *Staphylococcus aureus*	EtOH extract showed antibacterial and antifungal activities against all the strains tested, IZ 1–11 mm depending on the microorganism and MIC 0.5–1.0 μg/mL.	[27]
*V. album*	Plaque assay	Human parainfluenza virus type 2 (HPIV-2)	EtOH extract (25 µg/mL) did not affect the growth rate or viability in VERO cells and was inactive in HPIV-2 plaque formation on Vero cells.	[32]
*V. album*	Fungal decay test in wood	*Coniophora puteana*	MeOH extract at 18,75% showed the lowest wood weight loss (7.97%).	[103]
*V. articulatum*	Disc diffusion assay	*Esherichia coli*, *Pseudomonas aeruginosa*, *Staphylococcus aureus*	EtOH extract (200 µg/mL) presented inhibitory activity against the microorganisms tested, IZ 9–20 mm depending on the microorganism evaluated.	[107]
*V. articulatum*	Disc diffusion assay, broth micro-dilution assay for MIC and MBC	*Bacillus cereus*, *Bacillus subtilis*, *Esherichia coli*, *Pseudomonas aureugenosa*, *Staphylococcus aureus*, *Staphylococcus typhi*	MeOH extract (50–2000 mg/disc) inhibited *Staphylococcus aureus* and *Esherichia coli* (MIC of 728 and 920 µg/mL, respectively)*,* with MBC of 1456 µg/mL and 1840 µg/mL and IZ 11.4 ± 0.05 and 10.1 ± 0.06 to *Staphylococcus aureus* and *Esherichia coli,* respectively.	[108]
*V. capense*	Disc diffusion assay	*Candida albicans*, *Staphylococcus aureus*, *Pseudomonas aeruginosa*	MeOH extract (40µL) showed inhibition only on *S. aureus* growth with IZ 12.8 mm	[115]
*V. monoicum*	Disc diffusion assay	*Esherichia coli*, *Salmonella paratyphi*, *Salmonella typhi*, *Shigella dysenteriae*, *Staphylococcus aureus*, *Staphylococcus epidermis*	EtOH extract at 250 and 500 µg/disc presented an tibacterial activity against all tested bacteria with IZ of the 6.35 to 9.10 mm and 10.25 to 13.00 mm, respectively.	[132]
*V. rotundifolium*	Micro-dilution assay	*Staphylococcus aureus*, *Enterococcus faecalis*, *Pseudomonas aeruginosa*, *Escherichia coli*	MeOH extract had MIC values range from 0.31 to 2.5 mg/mL.	[136]

IZ: inhibition zone; MIC: minimum inhibitory concentration; MBC: minimum bactericidal concentration; CFU: colony forming unit.

**Table 3:** *Viscum* sp. in vitro activities—antiparasitic and insecticide.

Species	Assay	Microorganism	Main Results	Authors
*V. album*	Movement of flagella and undulating membranes	Metronidazole-resistant *Trichomonas vaginalis*; metronidazole-sensitive *Trichomonas vaginalis*	EtOH fractions (1.25–10 mg/mL) presented MLD (minimum lethal dose) varying from 5 to 10 mg/mL.	[33]
*V. album*	Absolute deterrence coefficient and toxicity to Larvae	*Thaumetopoae solitaria*	EtOH extract (1000 mg) showed antifeedant (36.71) effect. Toxicity effect reached the LC_50_ value of 60%. Thus, it can be used as a cheaper alternative for chemical pesticides and diminish environmental pollution.	[34]
*V. congolensis*	Petri dish with extracts	*Alma emini* worms	EtOH extract (19 mg/mL) reached 100% of mortality after 24 h. LC_50_ (1.65 mg/mL).	[126]

**Table 4:** *Viscum* sp. in vitro activities—cytotoxic/cytostatic.

Species	Assay	Cell Line	Main Results	Authors
*V. album*	MTT	Human breast adenocarcinoma (MDA-MB-231)	MeOH extract (50–1000 mg/L) presented IC_50_ of 12.75 mg/L	[13]
*V. album*	MTT; Apoptotic activity; FITC Annexxin V and propidium iodide (PI)	Murine melanoma (B16F10), human chronic myelogenous leukaemia (K562), monkey kidney (MA-104)	EtOH extracts presented a dose-response activity (1–5% *v*/*v*), with higher potential against B16F10 and K562 than MA-104. Annexin-V/FITC demonstrated early and late apoptotic markers on cancer cells.	[35]
*V. album*	MTT; Apoptotic activity; caspase-3 assay	Rat glioma (C6)	MeOH extract presented IC_50_ = 270 µg/mL on C6 cells. It decreased the expression of Hsp27 (73%), 14-3-3β (124%), 14-3-3γ (23%) and 14-3-3ζ (84%), thus downregulating the expression of chaperone proteins and inducing apoptosis via caspase-3.	[36]
*V. album*	MTT	Human cervical carcinoma (HeLa)	MeOH extracts (host tree *Tilia argentea*, *Acer campestre* and *Robinia pseudoacacia*) decreased the viability of HeLa cells in a dose-dependent manner (IC_50_ 93, 165, 85 µg/mL, respectively). Extracts from *Robinia* and *Tilia* host trees completely prevented nuclear and mitochondrial DNA damage.	[37]
*V. album*	MTT, BrdU immunoassay, lactate dehydrogenase (LDH) toxicology assay	Human colon adenocarcinoma (LS180), Human colon epithelial (CCD 841 CoTr)	MEOH extract had antiproliferative activity in a dose-dependent manner, reaching 30% of reduction at 100µg/mL. IC_50_ were 1 mg/mL, 164 µg/mL, 202 µg/mL, from host tree *Pinus sylvestris*, *Tilia cordata*, *Populus nigra*, respectively. BrdU assay demonstrated no effect on the DNA synthesis. LDH test showed nontoxic effect, indicating protective properties.	[39]
*V. album*	MTT	Human breast adenocarcinoma (MCF-7), human hepato- cellular carcinoma (HepG2)	EtOH and MeOH extracts (15–75 mg/mL) presented antiproliferative activity in a dose dependent manner.	[40]
*V. album*	MTT	Human hepatocyte carcinoma (HepG-2)	MeOH extract (50–1000 µg/mL) decreased cell viability in a concentration-dependent manner (CC_50_ of 456.5 µg/mL)	[88]
*V. album*	MTT and cytometry	Human acute lymphoblastic leucemia (Molt-4), Mouse sarcoma (Yoshida)	EtOH extract of *V. album* from *Abies alba* presented IC50 of 0.07 ± 0.01% *v*/*v* and 0.05 ± 0.03% *v*/*v* (Molt-4 and Yoshida, respectively). Necrotic potential of *Abietis*, *Malus* and *Quercus* was observed.	[90]
*V. album*	MTT	Breast cancer (MB-MDA 435)	MeOH extract (50–1000 mg/L) presented IC_50_ of 172 mg/L, presenting significant antiproliferative property	[104]
*V. angulatum*	MTT	Human breast adenocarcinoma (MDA-MB-231)	MeOH extract (0.1–100 µg/mL) from the host tree *O. dioica* decreased cell proliferation in a dose dependent manner (LC_50_ 79.33 µg/mL) while the one from *F. indica* host tree presented no activity (LC_50_ 500.82 µg/mL).	[106]
*V. coloratum*	MTT	Human ovarian carcinoma (HO-8910), human hepatocarcinoma (SMMC-7721), urinary bladder carcinoma (T24), human liver carcinoma (HepG2), human glioma (SHG)	Five fractions from EtOH extract (IC_50_ 18.6–465.3 µg/mL for HO-8910; IC_50_ 32.1–904.9 µg/mL for SMMC-7721) and isolated compounds (IC_50_ 6.7–28.3 µg/mL for HO-8910; IC_50_ 12.1–50.0 µg/mL for SMMC-7721) were cytotoxic. Isolated 3-*epi*-betulinic acid, oleanolic acid and erythrodiol exhibited the most significant activity.	[118]
*V. coloratum*	DNP-HAS and EZ-Cytox assay	Basophilic leukaemia cell line (RBL-2H3)	EtOH extract was not cytotoxic to IgE-sensitized cells (RBL-2H3).	[14]
*V. coloratum*	EZ-Cytox assay	Human colorectal adenocarcinoma (Caco-2)	EtOH extract (100–200 µg/mL) was not cytotoxic to the cell-derived inflammatory mediator (MDIM)-activated Caco-2 cells.	[119]
*V. cruciatum*	Bradford colorimetric method	Human laryngeal carcinoma (HEp-2)	MeOH extract (30 µg/mL) exhibited moderate cytostatic activity compared to 6-mercaptopurine positive control (36.04%).	[127]
*V. cruciatum*	Sulforhodamine B	Human renal adenocarcinoma (TK-10), human breast adenocarcinoma (MCF-7), human melanoma (UACC-62)	Hirsutanone isolated from MeOH extract presented GI_50_ values of 6.8, 1.9 and 4.8 µg/ml, when tested for TK-10, MCF-7 and UACC-62, respectively. Etoposide was used as a positive control (GI_50_ values for TK-10 cells, MCF-7 cells and UACC-62 cells were 8.1, 0.33 and 0.97 mg/ml, respectively).	[128]
*V. cruciatum*	Sulforhodamine B	Breast cancer cell line MCF-7	MeOH extract after 72 h presented IC_50_ 73 µg/mL.	[23]
*V. orientale*	Brine shrimp lethality	Brine shrimp nauplii	MeOH extract (10–320 µg/mL) showed potent cytotoxicity with LC_50_ of 21.63 μg/mL.	[134]

**Table 5:** *Viscum* sp. in vitro activities—cell migration and metalloproteinases inhibition.

Species	Assay	Cell Line	Main Results	Authors
*V. album*	Metalloproteinase inhibition	IL-1β-stimulated chondrocyte cells (SW1353)	EtOH extract inhibited MMP-13 expression at 20–200 μg/mL (64.3%, 70.3% and 80.0% inhibition at 50, 100 and 200 μg/mL, respectively)	[95]
*V. coloratum*	Wound healing and transwell migration	MDIM-stimulated chondrocyte cells (SW1353)	EtOH extract reduced cell migration and inhibited the expression, secretion and/or activity of MMP-1, MMP-3 or MMP-13 in MDIM-stimulated SW1353 cells demonstrating anti-osteoarthritic properties	[14]

**Table 6:** *Viscum* sp. in vitro activities—antiplatelet and antihypertensive.

Species	Assay	Material	Main Results	Authors
*V. album*	Prothrombin time (PT) and activated partial thromboplastin time (PTT)	Human citrated whole blood	MeOH extract from *V. album* leaves from olive and almond host trees presented prolongation of prothrombin time (PT) and activated partial thromboplastin time (aPTT), important indicators of coagulation.	[25]
*V. album*	Inhibition of platelet aggregation and effects on arachidonic acid	Human citrated whole blood	EtOH isolated phenylpropanoids (0.001–1.0 µM) inhibited ADP-induced platelet aggregation. Arachidonic acid metabolism and biosynthesis of leukotrienes were not affected. Two di-glycosides (10 µM) inhibited 20–25% leukotriene B4 release. It suggests antitumor activity related to inhibition of protein kinase C by phenylpropanoids.	[21]
*V. album*	Platelet aggregation	Rabbits and human platelets	EtOH extract (1–10 mg/mL) exhibited a dose dependent inhibition on platelet aggregation (IC_50_ 2.3–3.4 mg/mL).	[41]
*V. cruciatum*	Platelet aggregation	Human platelet rich plasma	EtOH extract (0.3, 0.6 and 1.2 mg/mL) inhibited the adrenaline (15%, 35%, 59%) and ADP (33%, 41%, 75%) induced human platelet aggregation.	[129]
*V. triflorum*	Angiotensin-converting enzyme (ACE) inhibitory activity	Rabbit lung ACE	EtOH extract presented no significant activity on ACE inhibition when compared to captopril positive control IC_50_ 12.0 ± 2.6 nM.	[140]

**Table 7:** *Viscum* sp. in vitro activities—anti-inflammatory effects.

Species	Assay	Material/Cell	Main Results	Authors
*V. album*	Inhibitory biosynthesis of IL-1α, IL-1β, TNF-α	Human whole blood	EtOH extract (1–30 µg/mL) exhibited none to insignificant inhibitory activity on cytokines	[43]
*V. articulatum*	Hypotonicity-induced hemolysis	Human whole blood	MeOH extract (0.5–5.0 mg/mL) possess anti-inflammatory activity (41.6–32.0%) in a dose dependent manner, comparable to the standard drug (indomethacin).	[109]
*V. coloratum*	β-hexosaminidase assay, enzyme-linked immunosorbent assay for TNF-α and IL-4, enzyme immunoassay for PGD2 and LTC4	Mast cell line from rat basophilic leukemia RBL-2H3 cells	EtOH extract inhibited degranulation (IC_50_ 93.04 μg/mL), production of IL-4 (IC_50_ 73.28 μg/mL), TNF-α (IC50 50.59 μg/mL), PGD2 and LTC4 and the activation of the FcεRI signalling cascade in IgE/Ag-activated RBL-2H3 cells.	[14]
*V. coloratum*	Mast cell-mediated colitis	Mast cell-derived inflammatory mediator (MDIM)-activated Caco-2 cells	EtOH extract (100–200 µg/mL) suggested multiple targets, such as mast cells, macrophages, neutrophils, MMP-2, MMP-9, Jak2 and STAT3 for anticolitic activity.	[119]

**Table 8:** *Viscum* sp. in vitro activities—hypoglycemic/hypolipemic activity.

Species	Assay	Material	Main Results	Authors
*V. album*	Anti-glycation and superoxides assays		MeOH extract (IC_50_ 199.8 µM) exhibited significant (72.5%) antiglycation activity, as well as six isolated compounds, inhibiting advanced glycation end products formation	[45]
*V. album*	α-amylase and α-glucosidase type IV inhibitory activities	Porcine pancreatic α-amylase, type IV α-glucosidase enzyme from *B. stearothermophilus*	EtOH extract (100–3000 µg/mL) of subsp. *album* presented 1.8–8.7% of α-amylase inhibition, while spp*. austriacum* (300–3000 µg/mL) presented 2.6–44.3% of inhibition. It possessed dose-dependent activity on α-glucosidase, with IC_50_ (mg/mL) of 0.7962 and 0.6653, respectively. Thus, it can ameliorate hyperglycaemia in type 2 diabetics	[46]
*V. album*	Pancreatic lipase and phosphodiesterase (PDE) inhibitory activities		EtOH extract presented inhibitory activity on lipase (IC_50_ 33.32 µg/mL) and on phosphodiesterase (IC_50_ 35.15 µg/mL)	[47]
*V. articulatum*	α-Amylase inhibitory activity		EtOH extract (0.2–1 mg/mL) inhibited α-amylase from 25 to 83% (IC_50_ 53.79 µg/mL). Thus, it showed anti-diabetic activity	[110]
*V. schimperi*	Glycation of albumin and its endproducts, protein aggregation using thioflavin T		MeOH extract (3–330 mg/mL) decreased advanced glycation endproducts (AGE) and protein aggregation (PA). Fractions from MeOH extract showed different inhibitory activity on AGE formation and PA	[137]

**Table 9:** *Viscum* sp. in vitro activities—cellular antioxidant effect.

Species	Assay	Main Results	Authors
*V. album*	Hydroxyl scavenging activity (HRSA) using deoxyribose method, superoxide radicals (SRSA) using xanthine oxidase, lipid oxidation using thiobarbituric acid reactive substance (TBARS)	EtOH extract (0.5 mg/mL) presented 40.19% of inhibitory activity for HRSA and 30.05% for SRSA. The extract reduced TBARS after 14 days of storage.	[31]
*V. album*	SOD, CAT, glutathione-Stransferase (GST), GR	MeOH extract (50–1000 mg/L) presented IC_50_ of 12.75 mg/L. SOD, Catalase, Glutathione S-Transferase, Glutathione Reductase activities increased in the presence of the extract, reducing superoxide and hydrogen peroxide radicals accumulated in the MDA-MB-231	[13]
*V. album*	Oxidative stress and intracellular ROS lever by H_2_O_2_ induction and DCFH-DA	Extracts from *Robinia* and *Tilia* host trees completely prevented nuclear and mitochondrial DNA damage under stress conditions, while extract from Acer was completely effective for nuclear DNA damage but only half-effective for mitochondrial DNA damage.	[37]
*V. album*	Lipid peroxidation using malondialdehyde (MDA)	EtOH extract (0.5 µg/mL) had protective effect against lipid peroxidation and DNA repairing. Thus, it is promising as antioxidant, anti-mutagenic and DNA repair-inducing properties	[42]
*V. album*	5-Lipoxygenase and acetylcholinesterase inhibitory activities	EtOH extract inhibited lipoxygenase and acetylcholinesterase with IC_50_ of 0.236 ± 0.030 and 1.712 ± 0.080 mg/mL, respectively.	[48]
*V. album*	Tryosinase and superoxide dismutase activity	EtOH extract presented 62.88% of inhibitory effect on tyrosinase and 53.55% of superoxide dismutase inhibition	[49]
*V. album*	SOD, (CAT), glutathione-S-transferase (GST), GR	SOD, Catalase, Glutathione S-Transferase and Glutathione Reductase activities varied at 100 mg/L after the periods evaluated (24, 48 and 72 h).	[104]

**Table 10:** *Viscum* sp. in vivo activities—hypoglycemic.

Investigation	Animal Model	Intervention	Main Results	Authors
Antidiabetic and hypoglycemic activity in streptozotocin-induced diabetic rats.	Male Wistar rats	50 mg/kg and 100 mg/kg b.w. of the extract administered for 4 h or 100 mg/kg the extract administered daily for 3 consecutive weeks.	Hypoglycemic effect within 4 h of administration was 41% (50 mg/kg) and 49% (100 mg/kg) at 2 h. Extract at 100 mg/kg for 3 weeks decreased LDL serum level from 1137.0 to 728.4 (IU/L) in streptozotocin-diabetic rats.	[50]
Antidiabetic activity in streptozotocin-induced diabetic rats by fasting blood sugar.	New Zealand white albino rats	250, 500, 750 and 1000 mg/kg b.w.	750 mg/kg b.w. decrease fasting blood glucose level in normal as well as in streptozotocin-induced diabetic rats similar with the glibenclamide control.	[51]
Antidiabetic activity in partial pancreatectomized rats.	Male Sprague-Dawley rats	0.6% of EtOH extract in diet for 8 weeks.	EtOH extract enhanced glucose-stimulated insulin secretion and β-cell proliferation in diabetic partial pancreactomized rats.	[52]
Antidiabetic activity was assessed through fasting blood glucose level, insulin levels and area under the curve in oral glucose tolerance test.	Male Wistar rats	75 and 150 mg/kg b.w. of the lyophilized extract was administered in an oral dose.	MeOH extract and organic subfractions presented a significant antihyperglycemic activity after 4 weeks of daily doses. Insulin levels increased in a dose dependent manner and all tested fractions produced reduction in AUC of glucose concentration.	[138]
Antihyperglycemic and hypolipidemic effect by effect of extract on plasma glucose level, oral glucose tolerance test, plasma insulin level, muscle and liver glycogen and plasma lipid profile.	Male Wistar rats	500 mg/kg b.w. of the extract given orally by gavage as single daily treatments for 4 weeks.	Antihyperglycemic activity was observed by maximum reduction in blood glucose level of 37%.	[139]

**Table 11:** *Viscum* sp. in vivo activities—hypolipemic.

Investigation	Animal Model	Intervention	Main Results	Authors
Hypocholesterolemic activity through measurement of serum total cholesterol, triglyceride, HDL-C, LDL-C concentrations in mice fed with cholesterol-rich diet.	Male Swiss albino mice	100 mg/kg b.w. of the extract after suspending in a mixture of distilled H_2_O and 0.5% sodium carboxymethyl cellulose were used in an orally gastric gavage.	EtOH extract reduced the serum cholesterol concentration in 59.1%, increased the serum HDL in 46.7%, decreased 83.0% the serum LDL-C concentration and decreased the serum triglyceride concentration in 60.7% without inducing any gastric damage.	[54]
Hypolipemic activity in atherogenic diet induced hyperlipidaemic model in mice was performed by cholesterol, triglyceride, low-density lipoprotein and high-density lipoprotein in blood serum of albino mice.	Swiss albino mice	Extract at 200 mg/kg/day in oral suspension 0.2% *w*/*v* of the gum acacia powder in distilled water.	EtOH extract did not present activity on reduction of total cholesterol, triglyceride, low-density lipoprotein, high-density lipoprotein and atherogenic index when compared with the control.	[111]
Antihyperglycemic and hypolipidemic effect by effect of extract on plasma glucose level, oral glucose tolerance test, plasma insulin level, muscle and liver glycogen and plasma lipid profile.	Male Wistar rats	500 mg/kg b.w. of the extract given orally by gavage as single daily treatments for 4 weeks.	Hypolipemic effect was demonstrated by significant reductions in plasma total cholesterol (32.6%), in triglyceride (32.2%) and in low-density lipoprotein cholesterol (27.2%) and an increase in high-density lipoprotein-cholesterol of 171.5%.	[139]

**Table 12:** *Viscum* sp. in vivo activities—anticancer.

Investigation	Animal Model	Intervention	Main Results	Authors
Antimetastatic activity in Wistar rats.	Female Wistar rats	Mixture of *V. album* L. + *Abies alba* (136 mg/kg b.w. of extract in the first week, 271 mg/kg b.w. of extract for the 2 week and 406 mg/kg b.w. of extract for the last 3 weeks intraperitoneally.	The *V. album* L. + *Abies alba* extract reduced the metastatic locations almost by 77%.	[41]
Anticancer activity in Ehrlich ascitic carcinoma model.	White Swiss female mice	EtOH extract at 50 mg dry substance/kg b.w. in the days 1, 3 and 6 intraperitoneally.	Doxorrubicin and *V. album* association provided anti proliferative effect when compared to doxorubicin alone reducing difference in body weight, Ehrlich ascitic volume and cellular concentration.	[55]
Anticancer effect evaluated by Ehrlich ascites carcinoma in mice.	White Swiss female mice	50 mg/kg b.w. of the extract restored with sterile saline solution, equivalent to 18 µL of EtOH tincture, i.p.	Extract at 50 mg/kg + doxorubicin reduced the Ehrlich ascite carcinoma volume from 8.43 (control) to 0 when compared to untreated group, the catalase activity from 3.5 (control) to 1.5 mU/mL and the xanthine oxidase activity from 0 to approximately 4 mU/mL.	[56]

**Table 13:** *Viscum* sp. in vivo activities—hypotensive.

Investigation	Animal Model	Intervention	Main Results	Authors
Hypotensive activity on values of arterial blood pressure by direct method in the left carotid artery.	Male and female Wistar rats	3.33 × 10^−5^, 1 × 10^−4^, 3.33 × 10^−4^, 1 × 10^−3^ mg/kg of the extract were administered through right outer jugular vein.	EtOH extracts presented dose dependent response and the maximal reduction was observed at 1 × 10^−3^ mg/kg with arterial blood pressure reduction of the 23.56 mmHg with effect via muscarine receptors.	[15]
Diuretic, saluretic and natriuretic effects by Na^+^ and Cl^−^ excretions.	Male Wistar rats and Swiss albino mice	100, 200 and 400 mg/kg b.w. of the MeOH extract dissolved in water and administered orally.	The extract at 400 mg/kg had a diuretic index (volume in test group/volume in control group) in 24 h of 2.76 and increased saluretic (Na^+^ + Cl^−^) at 168 and natriuretic index (Na^+^/K^+^) at 2.20.	[105]
Diuretic, saluretic and natriuretic effects by Na^+^ and Cl^−^ excretions.	Male Wistar rats and Swiss albino mice	100, 200 and 400 mg/kg b.w. of the MeOH extract dissolved in water and administered orally.	400 mg/kg of the extract had a diuretic index (volume in test group/volume in control group) at 24 h of 3.00 and increased saluretic (Na^+^ + Cl^−^) at 272 and natriuretic index (Na^+^/K^+^) at 2.16.	[112]
Antihypertensive activity against Nω-nitro-L-arginine methyl ester induced hypertension by blood pressure and heart rate; urine volume and urine sodium/potassium; serum creatinine; serum lipid estimation.	Male Wistar rats	200 or 400 mg/kg/day of the extract orally.	MeOH extract prevented progression of hypertension in rats produced by chronic administration of Nω-nitro-L-arginine methyl ester, which may be due to its diuretic, nephroprotective, hypolipemic and antioxidant effects.	[113]

**Table 14:** *Viscum* sp. in vivo activities—analgesic and anti-inflammatory.

Investigation	Animal Model	Intervention	Main Results	Authors
Anticolitic effect in induced colitis for 8 days by Dextran Sodium Sulfate (DSS).	Male C57BL/6 mice	0–200 mg/kg −100 μL orally once a day.	Extract attenuated the body weight loss, reduced the scores of Disease Activity Index (DAI), suppressed enterorrhagia and colonic oedema in DSS-treated mice.	[119]
Analgesic activity by tail immersion method and anti-diarrhoeal activity by Castor oil-induced diarrhoea method.	Male and female Swiss-albino mice	For the analgesic activity, the tail immersion test in hot water was performed after treating the animals via a gastric tube. For the anti-diarrhoea activity, the mice were treated orally after being induced to diarrhoea.	EtOH extract of *V. monoicum* exhibited analgesic activity through central nervous system in dose dependent manner. EtOH extract of *V. monoicum* showed anti-diarrheal activity decreasing defecation in a dose dependent manner.	[132]
Anti-inflammatory activity by Carrageenan-induced oedema and liver-protective effects in CCl_4_-induced hepatotoxicity.	Male Wistar albino rats	100 and 300 mg/kg b.w. of the extract subcutaneously.	EtOH extract promoted anti-inflammatory activity reducing the paw oedema to indomethacin. Extract was not effective in protecting the liver against CCI_4_-induced damage.	[133]

**Table 15:** *Viscum* sp. in vivo activities—Neuropharmacological.

Investigation	Animal Model	Intervention	Main Results	Authors
Antianxiety, anti-depressant, hypnotic, anti-stress and analgesic activities were performed using elevated plus maze (EPM), forced swim test (FST), thiopentone sodium-induced sleeping assay, cold swim test and tail immersion test, respectively. Behavior activity was preformed using open field test.	Male and female Laca mice	50–400 mg/kg b.w. of the extract orally.	50–400 mg/kg of MeOH extract exhibited significant antianxiety activity, increasing the number of entries and time spent in open arms of EPM; reduced duration of immobility in antidepressant activity by despair swim and the rearing and crossings in open field test. Higher doses increased duration of sleeping mice and reduced time spent by mice in immobile state activity and analgesic activity.	[57]
Monoamine oxidase A and monoamine oxidase B activity	*Galleria mellonella* larvae	1.0%, 2.5% and 5.0% EtOH extract in diet	All concentrations evaluated inhibited both MAO-A and MAO-B.	[96]
Anticonvulsant activity induced in mice with pentylenetetrazole, bicuculline and N methyl-DL-aspartic acid.	Male and female albino mice	Intraperitoneal injection at 50–100 mg/kg b.w. of the extract in a physiological saline solution.	MeOH extract protected the mice against pentylenetetrazole- and bicuculline-induced tonic seizures but did not significantly alter N-methyl-DL-aspartic acid-induced tonic seizures, suggesting its antiepileptic effect.	[115]
Behavior and antianxiety by open field exploratory, hypnotic/sedative effect by pentobarbitone-induced sleep.	Swiss albino mice	100, 200 or 400 mg/kg b.w. of extract orally.	Animals treated with a dose of 400 mg/kg of MeOH extract decreased the number of total crossings. The extract demonstrated a dose dependent increase in pentobarbitone induced sleep.	[117]
Anti-nociceptive and central nervous system activity through acetic acid and formalin-induced pain models, respectively, and cross and open field test behavior profiles.	Male and female Swiss albino mice	300 or 500 mg/kg b.w. of the extract orally.	In the acetic acid induced writhing test, extract produced 88.8% of writhing inhibition at 500 mg/kg of body weight. Extract has both peripheral and neurogenic anti-nociceptive and CNS depressant activities.	[135]

**Table 16:** *Viscum* sp. in vivo activities—toxicity.

Investigation	Animal Model	Intervention	Main Results	Authors
Protective effects against cyclophosphamide-induced cardiotoxicity, urotoxicity and genotoxicity through anti-oxidative stress and -inflammation in the heart and bladder and chromosomal damage in the bone marrow.	Male Swiss albino mice	250 mg/kg b.w./day of the extract were administered orally by gastric gavage.	Antioxidant enzymes such as superoxide dismutase, catalase and glutathione peroxidase, Glutathione-S-transferases and mitotic index were restored to near normalcy as compared to the control group. Lipid peroxidation in heart and bladder were reduced by extract when compared to the cyclophosphamide group.	[58]
Anti-cytogenotoxic effects of pre-treatment with *V. album* extract on methotrexate-induced chromosomal aberrations.	Male Swiss albino mice	Extract by oral gavage at 250 mg/kg b.w./day for 10 days.	MeOH extract had a protective effect against methotrexate-induced cyto-genotoxicity in mouse bone marrow decreasing chromosomal aberrations of 96.40 ± 8.25 to 59.20 ± 1.65 in relation to the control.	[59]
Acute toxicity testing according to the oral administration method.	Male Balb/c mice	EtOH extract up to 5000 mg/kg body weight orally.	No animal deaths were observed in the study.	[94]
Protective effect against chlorpyrifos-induced hepatotoxicity.	Male Wistar albino rats	EtOH extracts 350 mg/kg b.w. intraperitoneally.	The extract recovered the antioxidative system parameters and alleviated some histopathological changes caused by chlorpyrifos.	[97]
Protective effect against tetrachloride (CCl_4_)-induced acute/chronic liver injury.	Male Wistar rats	MeOH extract 300 mg/kg b.w. orally in single dose	The extract decreased ALT and AST enzymes increased by CCl_4._	[101]
Acute toxicity testing according to the oral administration method.	Swiss albino mice	MeOH extract at 5–2000 mg/kg b.w. suspended in 0.5% carboxymethyl cellulose	The extract was safe up to 2000 mg/kg body weight.	[117]
Acute toxicity testing according to the intragastric administration method.	Kunming mice of both sexes	Ethanolic extract.	EtOH extract presented an acute toxicity of the LD_50_ 7.67 g/kg.	[118]

**Table 17:** *Viscum* sp. in vivo activities—other activities.

Investigation	Animal Model	Intervention	Main Results	Authors
Bleeding time test by tail transection.	Rats	125, 250 and 500 mg/kg of the extract b.w. given orally once a day for 12 days.	500 mg/kg of the Korean and European MeOH extract prolonged the bleeding time 185.6% and 175.7% respectively compared to the control.	[60]
Effects of *V. album* on cardiac function through the nitric oxide pathway in isoproterenol-induced heart failure rats evaluated by echocardiographic and biochemical evaluation.	Male Wistar albino rats	Concentrated extract reconstituted in 0.9% NaCl and administered 250 mg/kg/day orally.	MeOH extract improved: left ventricular diameters, ejection fraction, serum N-terminal pro b-type natriuretic peptide) levels and histopathological changes. Attenuation of increased levels of nitric oxide and nitric oxide synthase. The levels of high-sensitivity C-reactive protein were lower in the *V. album* group compared to the controls.	[61]
Pharmacokinetic studies	Male and female Wistar rats	Injection of 13.2 mg·kg^−1^ of the homoeriodictyol-7-O-b-D-glucopyranoside isolated from *V. coloratum* via the tail vein.	The method was successfully applied to the pre-clinical pharmacokinetic study of homoeriodictyol-7-O-b-D-glucopyranoside with AUC of e 16.04 ± 3.19 µg.h.mL^−1^. T1/2α and t1/2β were 0.06 ± 01 h and 1.27 ± 0.31 h, respectively. Homoeriodictyol-7-O-b-D-glucopyranoside was cleared from the blood and mainly distributed to the liver and small intestine.	[63,120]
Growth performance	Rainbow trout (Oncorhynchus mykiss)	T1 (0.5%); T2 (1.5%); T3 (2.5%); and T4 (4%) MeOH supplemented in diet.	Here, 1.5 and 2.5% of extract in diet increased protease activity and serum antioxidant enzyme activities. These concentrations decreased serum activities of hepatic enzymes. The highest serum lysozyme and total Ig values were observed in the 2.5% of the extract in diet.	[100]

**Table 18:** *Viscum* sp. ex vivo studies.

Investigation	Main Results	Authors
Vascular effects in noradrenaline-contracted rat aortic rings.	*n*-BuOH fraction produced a contractile response in noradrenaline-contracted rat aortic rings.	[63]
Vascular effects on isolated noradrenaline-contracted rat aortic segments.	EtOH extract contained marked vasodilator activity especially from cherry, quince and acacia host trees.	[62]
Gut inhibitory and stimulatory effects by in rabbit jejunum and guinea-pig ileum respectively.	Spasmogenic effect was observed with a concentration-dependent contractile effect in guinea-pig ileum at 5–10 mg/mL of the extract. Spasmolytic effect was demonstrated to relax the spontaneous and K^+^ (80 mM)-induced contractions of isolated rabbit jejunum, with EC_50_ values of 0.66 and 0.55 mg/mL, respectively.	[129]
Antispasmodic and relaxant activity in smooth muscle evaluated in isolated rabbit jejunum and in rabbit aortic rings.	Crude MeOH extract inhibited spontaneous and high K^+^-induced contractions in rabbit jejunum. The extract showed a partial relaxation against high K^+^ (80 Mm) and phenylephrine (1 μM)-induced contractions in isolated rabbit aorta rings.	[65]
Cardioprotective activity in myocardial ischemia and reperfusion injury in rats.	5 mg/L of the MeOH extract reduced 53.2% in mean infarct size compared to control hearts.	[64]

## Data Availability

The data summary is contained within the article.

## Supplementary Materials

The following supporting information can be downloaded at: https://www.mdpi.com/article/10.3390/plants12091811/s1. Table S1: Secondary metabolite total content and antioxidant capacity in alcoholic *Viscum* sp.; Table S2: Preliminary phytochemical analysis in alcoholic *Viscum* sp.; Table S3: Fatty acids in alcoholic *Viscum* sp.; Table S4: Carbohydrates in alcoholic *Viscum* sp.; Table S5: Phenolic acids and their derivatives in alcoholic *Viscum* sp.; Table S6: Flavonoids and their derivatives in alcoholic *Viscum* sp.; Table S7: Terpenoids and their derivatives in alcoholic *Viscum* sp.; Table S8: Miscellaneous in alcoholic *Viscum* sp.
