# Supplementary material for: A Scoping Review of Genus Viscum: Biological and Chemical Aspects of Alcoholic Extracts
Source: Plants (Basel). 2023 Apr 28;12(9):1811. doi: 10.3390/plants12091811 (PMC10181296; doi:10.3390/plants12091811)
Supplement: Supplementary file 1 [file plants-12-01811-s001.zip › plants-2296783-supplementary.pdf]

A Scoping Review of Genus *Viscum*: Biological and Chemical Aspects of Alcoholic Extracts - Supplementary material

Michelle Nonato de Oliveira Melo, João Vitor da Costa Batista, Evelyn Maribel Condori Peñaloza, Adriana Passos Oliveira, Rafael Garrett, Stephan Baumgartner, Carla Holandino

Table S1. Secondary metabolite total content and antioxidant capacity in alcoholic *Viscum* sp.

**Antioxidant activity**			
***Viscum* species**	**Total content or method for antioxidant capacity assay**	**Results**	**Author(s)**
Viscum album	Total phenolic content (TPC)	TPC: 19.43 mg gallic acid equivalent/g of dry weight	[29]
		TPC: between 170.71 and 443.99 mg gallic acid equivalent/mg of dry matter	[13]
		TPC: 4.78 to 10.43 mg gallic acid equivalent/g of dry extract	[39]
		TPC: 28.141±0.942 mg gallic acid equivalent/g of dry weight	[48]
		TPC: 5.86 ± 0.63 mg gallic acid equivalent/g of dry weight	[52]
		TPC: 182 mg tannic acid equivalent/100 g	[67]
		TPC: 1232.00 ±0.02 mg galic acid equivalent/g dry sample	[68]
		TPC: 6.33 ± 1.21 mg caffeic acid equivalent/g of dry plant	[69]
		TPC: 57.673 mg gallic acid equivalent/g of dry weight	[70]
		TPC: between 0.65 ± 0.19 and 0.40 ± 0.19 mg gallic acid equivalent/g fresh weight	[71]
		TPC: between 21.2 ± 2.0 and 35.8 ± 1.3 mg gallic acid equivalent/g of dry weight	[46,71]
		TPC: between 58.97 ± 0.009 and 51.96 ± 0.006 mg gallic acid equivalent/g of fresh matter depend on the host tree and part of the plant	[72]
		TPC: leaves between 31.32±0.008 to 65.30±0.002 mg galic acid equivalent/100g of fresh matter depend on the host tree<br>TPC: stems between 23.86±0.013 to 42.45±0.008 mg galic acid equivalent/100g of fresh matter depend on the host tree	[83]
		TPC: 39 ± 6.88 mg gallic acid equivalent/g dry weight plant material	[92]
		TPC: 13-19 mg gallic acid equivalent/g crude extract	[93]
		TPC: 94.38 ± 0.62 mg gallic acid equivalent/g extract	[97]

		TPC: 21.26 ± 0.003 (EtOH) and 23.51 ± 0.002 (MeOH) mg gallic acid equivalent/g extract	[98]
		TPC: 30.56 - 56.75 mg gallic acid equivalent/g dry extract	[99]
		TPC: 15 – 31 mg gallic acid equivalent/g dry extract	[102]
		TPC: 31.19 ± 0.60 mg gallic acid equivalent/g of dry weight	[46]
	Total proanthocyanidin content (TPAC)	TPC: 60.43 ± 3.38 mg catechin equivalent/g of extract	[31]
		TPC: 668.5 mg (+)-catechin equivalente/100 g of dry weight	[49]
	Total flavonoids content (TFC)	TFC: 36.38 ± 1.89 mg quercetin equivalent/g of extract	[31]
		TFC: 0.27 to 0.428 mg quercetin equivalent/g of dry extract	[39]
		TFC: 6.60 ± 0.55 mg quercetin equivalent/g of dry weight	[46]
		TFC: 5.998±0.215 mg quercetin equivalent/g of dry weight	[48]
		TFC: 0.54 ± 0.04 mmol/g dry matter 0.20 ± 0.02 μmol quercetin equivalent/g of dry matterial; 5.79 ± 0.24 μmol rutin equivalent/g of dry matter; 0.16 ± 0.02 μmol kaempferol equivalent/g of dry matterial	[52]
		TFC: 2.38 ± 0.31 mg rutin equivalent/g dry weight	[52]
		TFC: 9.72 ± 2.81 μg quercetin equivalent/g of dry plant	[69]
		TFC: 9.955 mg quercetin equivalent/g of dry weight	[70]
		TFC: 31.38 mg rutin equivalente/g of fresh material	[22]
		TFC: 3067 ± 17.2 mg quercetin equivalent/g dry weight plant material	[92]
		TFC: 3.38 - 2.61 mg rutin equivalents/ g crude extract	[93]
		TFC: 65.24 ± 0.23 mg rutin equivalents/ g extract	[97]
		TFC: 22.36 ± 0.45 (EtOH) 19.38 ± 0.35(MeOH) mg quercetin/g extract	[98]
		TFC: 10.11 – 16.90 mg quercetin equivalents/g dry extract	[99]
		TFC: 2.8 – 23 mg rutin equivalents/g dry extract	[102]
	Total carotenoids content (TCC)	TCC: between 0.22 ± 0.01 and 7.00 ± 0.15 mg β-carotene equivalent/g fresh weight	[71]
	DPPH (2,2-diphenyl-1-picrylhydrazyl) scavenging assay	DPPH: 4.66 ± 0.09 mol quercetin equivalent	[73]
		DPPH: 86.74%	[49]
		DPPH: IC_{50} 4.65 ±0.06 mg/mL	[68]
		DPPH: between 31.91± 0.00% to 77.19 ± 0.00 % higher for the leaves than for the stems, depend on the host tree and part of the plant	[83]
		DPPH: 70% inhibition	[67]
		DPPH: IC_{50} 2.650 ± 0.100 g of dry extract per g of DPPH	[70]
		DPPH: 82.99 ± 0.31 % inhibition	[46]

		DPPH: 7.20 ± 2.71 % inhibtion	[69]
		DPPH: IC_{50} 0.046 ± 0.061 mg/mL	[48]
		DPPH: IC_{50} 25.34 ± 3.8μg/mL (for olive *V. album*) and 15.37 ± 2.2μg/mL (for almond *V. album*)	[25]
		DPPH (%) clearance > 50% (three isolated substances)	[75]
		DPPH: IC_{50} 27.7 μM of gallic acid<br>DPPH: IC_{50} 222 μM of 3-(3'-carbomethoxypropyl) gallic acid	[74]
		DPPH: between 59,52 ± 4,51% to 95,12 ± 2,37 %	[76]
		DPPH: 20.23 % inhibition = equivalent 0.0388 mM Trolox, 0.1249 mM Ascorbic acid, 0.0366 Mm Chlorogenic acid	[91]
		DPPH: 87 – 93% inhibition depending on plant part and solvent (methanol or ethanol)	[93]
		DPPH: IC_{50} 7.28 ± 0.83 mg/mL	[97]
		DPPH: EC_{50} 2.14 - 4.35 mg dry extract/ mg DPPH	[99]
		DPPH: 14.8 – 49 mg equivalent Trolox/ g dry extract	[102]
	TEAC (Trolox Equivalent Antioxidant Capacity) assay	TEAC assay: leaves from *V. album* from *Fraxinus excelsior* (461.09 ± 0.11 mM Trolox equivalent/g of fresh matter) and from *Populus nigra* stems extract (306.68 ± 0.01 mM Trolox equivalent/g of fresh mater).	[72]
		TEAC assay: 0.96 ± 0.005 and 0.95 ± 0.106 mM Trolox equivalents/g fresh matter depend on the host tree and part of the plant	[83]
	FRAP (ferric reducing antioxidant power) assay	FRAP in mg galic acid equivalent-1 of the 1698.00 ±0.03	[71]
		FRAP: between 0.003 ± 0.10 and 0.14 ± 0.87 mg/L vitamin C equivalent /g of fresh leaves	[71]
	β-Carotene-linoleic acid assay	β-Carotene-linoleic acid assay: 82.23 % antioxidant activity	[29]
	SOD (Superoxide dismutase assay) assay	SOD: 53.55%	[49]
	SRSA (Superoxide anion radical scavenging activity) assay	SRSA: 30.05 ± 4.11 % inhibition	[31]
		SRSA: 16.66% of inhibition	[69]
		3-(4-acetoxy-3,5-dimethoxy)-phenyl-2E-propenyl-b-D-glucopyranoside and 4',5-dimethoxy-7-hydroxyflavanone showed antioxidant activity. SRSA = IC_{50} μM: 211.7 ± 7.0<br>SRSA = IC_{50} μM: 58.5 ± 3.0	[45]
	ORAC (Oxygen Radical Absorbance Capacity) assay	ORAC: 10.73 mM Trolox equivalents/g fresh matter (*V. album* from *Populus nigra* leaf extract).	[72]
		ORAC: < 10.73 ± 1.90 mM Trolox equivalents/g fresh matter depend on the host tree and part of the plant	[83]
	α-amylase / α-glucosidase inhibitory activity	α-amylase assay: 8.7±2.3 μg/mL and 44.3 ±4.1 μg/mL<br>α-glucosidase: IC_{50} (0.7962 mg/mL) and (0.6653 mg/mL)	[46]
		$ABTS^{•+}$: 50.2 ± 1.64 and 56.6 ± 0.45 mg/g	[46]

	ABTS$^{•+}$ (radical cation 2,2-azinobis-(3-ethylbenzothiazoline-6-sulphonic acid scavening activity) assay	ABTS$^{•+}$: IC_{50} 0.038 ± 0.079 mg/mL	[48]
		ABTS$^{•+}$: 41 – 75 mg Trolox equivalente/ g dry extract	[102]
	Reducing power capacity ($FeCl_3$ solution)	Reduce Fe^{+3} to Fe^{+2} dose-dependent pattern	[67]
		Reducing power capacity: 1.18 ± 0.01	[77]
	Chelation Power on (Fe^{2+}) ions activity assay	Fe chelation (II) assay: ca 60%	[67]
		Fe chelation (II) assay: 54.68 ± 2.77%	[69]
	FRSA (Free radical scavening activity) assay	FRSA: 34.01 ± 2.68 % inhibition	[31]
	HRSA (Hydroxyl radical scavening activity) assay	HRSA = 40.19 ± 1.61 % inhibition	[31]
		HRSA = 34.44% of inhibition	[69]
	H_2O_2 Hydrogen peroxide scavening activity assay	H_2O_2 = 0.37 % of inhibition	[69]
	DMPD (N, N-dimethyl-p-phenylendiamine radical scavenging activity) assay	DMPD 7.40 ± 1.38 % inhibition	[77]
	NO (Nitric oxide radical scavenging activity) assay	NO = 37.08 ± 1.05 % inhibition	[77]
		NO = 28.34% of inhibition	[69]
	PRAP (Phosphomolibdenum-reducing antioxidant power) assay	PRAP = 0.148 ± 0.024	[77]
	Metal-chelation capacity ($FeCl_2$) assay	Metal-chelation capacity = 83.79 ± 1.91 %	[77]
		Chelating metal ions IC_{50} 0.241±0.013 mg/mL	[48]
	AChE, BChE, and Tyrosinase activity assays	AChE: 9.78 ± 1.78 % inhibition; BChE: 26.30 ± 2.44 % inhibition; TYRO: 6.40 ± 1.32 % inhibition	[77]
	TBARS (Thiobarbituric acid reactive species) assay	TBARS: IC_{50}: 0.575 ± 0.120 mg/mL	[48]
	Thiobarbituric acid (TBA) methods	TBA: the results are more effective than Vitamin E	[76]
	XOD (Xanthine oxidase) assay	XOD (%) inhibitory activity > 50% (three isolated substances)	[75]
	Chemiluminescence	Chemiluminescence: 17.02 % inhibition	[73]
	FTM Ferric thiocyanate method	FTM: highest activity in comparison with Vitamin E	[76]
Viscum angulatum	Total phenolic content (TPC)	TPC: 41.80 μg gallic acid equivalent/mg of dry extract	[106]
		TPC: 284.1±7.643 μg gallic acid equivalent/mg	[105]
		TFC: 98.80 ± 6.445 μg quercetin equivalent/mg	[105]
	Total proanthocyanidin content (TPAC)	TPAC: 112.9 ± 5.939 μg epicatechin equivalent/mg	[105]
	DPPH (2,2-diphenyl-1-picrylhydrazyl) scavenging assay	DPPH: 57.88 + 0.66 %	[106]
Viscum articulatum	Total phenolic content (TPC)	TPC: 3.03 ± 0.02 g *p*-coumaric acid equivalente/100 g of dry material	[112]
	Total flavonoid content (TFC)	TFC: 1.75 ± 0.3 g quercetin equivalent/100 g of dry material	[112]
	Total proanthocyanidin content (TPAC)	TPAC: 1.37 ± 0.2 g catechin equivalent/100 g of dry material	[112]
	Total terpenoid content (TTC)	TTC: 19.5613 g (oleanolic acid+betulinic acid) equivalent/100 g of dry material	[112]

Viscum capitellatum	Total phenolic content (TPC)	TPC: 372.024 ± 09.572 mg gallic acid equivalent/100g dry weight	[116]
	Total phenolic contente (TPC)	TPC:47.84 ± 4.12 mg gallic acid equivalente /g dry extract	[117]
	Total flavonoid content (TFC) (flavanone; flavonol)	Total flavanone content: 51.917 ± 08.131 mg (±)-eriodictyol equivalent/100g of dry weight<br>Total flavonol content: 37.246 ± 01.787 mg quercetin equivalent/100g of dry weight	[116]
	Total flavonoid contente (TFC)	29.25 ± 2.20 mg quercetin equivalent/g dry extract	[117]
	Total carotenoids content (TCC)	TCC: 29.508 ± 02.467 mg β-carotene equivalent /100 g dry weight	[116]
	$ABTS^{•+}$ (radical cation 2,2-azinobis-(3-ethylbenzothiazoline-6-sulphonic acid scavening activity) assay	$ABTS^{•+}$: 407.109 ± 12.153	[116]
Viscum coloratum	Total phenolic content (TPC)	TPC: 11.14 ± 0.05 mg caffeic acid equivalent/g of dry weight	[14]
	Total flavonoid content (TFC)	TFC: 83.51 ± 0.56 mg quercetin equivalent /g of dry weight	[14]
	Total triterpene content (TTC)	TTC: ＞25.8 ± 1.1 mg oleanolic acid equivalent/g of dry weight	[118,130]
Viscum cruciatum	Total phenolic content (TPC)	TPC: 36.5 mg gallic acid equivalent/g of dry weight	[130]
	$ABTS^{•+}$ (radical cation 2,2-azinobis-(3-ethylbenzothiazoline-6-sulphonic acid scavening activity) assay	$ABTS^{•+}$: 590.5 μmol Trolox equivalent/ g of dry weight	[130]
Viscum orientale	Total phenolic content (TPC)	TPC: 73.4 mg gallic acid equivalent/g of dried weight	[135]
	Total flavonoids content (TFC)	TFC: 170.6 mg quercetin equivalent/g of dried weight	[135]
	DPPH (2,2-diphenyl-1-picrylhydrazyl) scavenging assay	DPPH: IC_{50} 6.63 μg/mL	[135]
Viscum rotundifolium	Total phenolic content (TPC)	TPC: 748.899 ± 3.170 mg gallic acid equivalent/g extract	[136]
	Total flavonoid content (TFC)	TFC: 2.869c ± 0.405 mg quercetin equivalent/g extract	[136]
Viscum schimperi	Total phenolic content (TPC)	TPC: 7 mg gallic acid equivalent/g dry weight	[137]
	Total flavonoid content (TFC)	TFC: 3 mg rutin equivalent/g dry weight	[137]
	DPPH (2,2-diphenyl-1-picrylhydrazyl) scavenging assay	DPPH IC_{50}: 10.0-22.0 μg/mL	[137]

Table S2. Preliminary phytochemical analysis in alcoholic *Viscum* sp.

Species	Preliminar phytochemical analysis	Author(s)
Viscum album	Positive for alkaloids, saponins, tannins, glycosides, flavonoids, terpenoids, triterpenoids, steroids, cardiac steroidal glycosides, emodin, anthraquinone glycosides, reducing sugar, coumarin, phenols and proteins.	[30,50,65,79]
Viscum articulatum	Positive for flavonoids, steroids, alkaloids, terpenoids and saponins.	[107,110]
Viscum congolensis	Positive for tannins, flavonoids and terpenes, as well as saponin, alkaloids, phenols and steroids.	[126]
Viscum cruciatum	Positive for alkaloids, coumarins, flavonoids, saponins, sterols, tannins, and terpenes.	[129]
Viscum monoicum	Positive for reducing sugars, tannins, saponins, gums, and alkaloids.	[132]
Viscum orientale	Positive for sugars, alkaloids, tannins and flavonoids.	[134]

Table S3. Fatty acids in alcoholic *Viscum* sp.

Fatty acids				
**Identified compounds**	**Chemical group**	**Species**	**Analytical method**	**Author(s)**
**10-Hydroxyoctadecenoic acid**	Fatty acid unsaturated	*Viscum schimperi*	UPLC- PDA-ESI-MS/MS	[138]
**Arachidic**	Fatty acid saturated	*Viscum album*	TLC; UV-light; IR-810; CD-J 500C; ^{1}H and ^{13}C-NMR; GLC; HPLC.	[80]
**Behenic acid**	Fatty acid saturated	*Viscum album*	TLC; UV-light; IR-810; CD-J 500C; ^{1}H and ^{13}C-NMR; GLC; HPLC.	[80]
**Cerotic acid**	Fatty acid saturated	*Viscum album*	TLC; UV-light; IR-810; CD-J 500C; ^{1}H and ^{13}C-NMR; GLC; HPLC.	[80]
**Dihydroxy-oleana-dien-oic acid**	Fatty acid unsaturated	*Viscum schimperi*	UPLC- PDA-ESI-MS/MS	[138]
**Hepracosainc acid**	n.d.	*Viscum album*	TLC; UV-light; IR-810; CD-J 500C; ^{1}H and ^{13}C-NMR; GLC; HPLC.	[80]
**Hydroxy-8,12-octadecadienoic acid**	Fatty acid unsaturated	*Viscum schimperi*	UPLC- PDA-ESI-MS/MS	[138]
**Hydroxy-octadecatrienoic acid**	Fatty acid unsaturated	*Viscum schimperi*	UPLC- PDA-ESI-MS/MS	[138]
**Lignoceric acid**	Fatty acid saturated	*Viscum album*	TLC; UV-light; IR-810; CD-J 500C; ^{1}H and ^{13}C-NMR; GLC; HPLC.	[80]
**Montanic acid**	Fatty acid saturated	*Viscum album*	TLC; UV-light; IR-810; CD-J 500C; ^{1}H and ^{13}C-NMR; GLC; HPLC.	[80]
**Stearic acid**	Fatty acid saturated	*Viscum album*	TLC; UV-light; IR-810; CD-J 500C; ^{1}H and ^{13}C-NMR; GLC; HPLC; GC-MS;	[80]

Table S4. Carbohydrates in alcoholic *Viscum* sp.

**Carbohydrates**				
**Identified compounds**	**Chemical group**	**Species**	**Analytical method**	**Author(s)**
**Arabinose**	Monosaccharides	*Viscum album*	GC-MS	[81]
**Galactose**	Monosaccharides	*Viscum album*	GC-MS	[81]
**Glucose**	Monosaccharides	*Viscum album*	GC-MS	[81]
**Inositol**	Sugar alcohols	*Viscum album*	GC-MS	[81]
**Mannose**	Monosaccharides	*Viscum album*	GC-MS	[81]
**Xylitol**	Sugar alcohols	*Viscum album*	GC-MS	[81]
**Xylose**	Monosaccharides	*Viscum album*	GC-MS	[81]

Table S5. Phenolic acids and their derivatives in alcoholic *Viscum* sp.

Phenolic acids and their derivatives – Hydroxycinnamic and Hydroxybenzoic acid derivatives

Identified compounds	Chemical group	Species	Analytical method	Author(s)
**1-*O*-caffeoylquinic acid**	Hydroxycinnamic acid derivatives	*Viscum schimperi*	UPLC- PDA-ESI-MS/MS	[92,137,138]
**3-*O*-caffeoylquinic acid**	Hydroxycinnamic acid derivatives	*Viscum album*	UPLC- PDA-ESI-MS/MS	[90,92]
**4-*O*-caffeoylquinic acid**	Hydroxycinnamic acid derivatives	*Viscum schimperi, Viscum album*	UPLC- PDA-ESI-MS/MS	[90,92]
**5-*O*-caffeoylquinic acid**	Hydroxycinnamic acid derivatives	*Viscum schimperi, Viscum album*	UPLC- PDA-ESI-MS/MS	[90,96,137,138]
**3-(3'-carbomethoxypropyl) gallic acid;**	Hydroxybenzoic acid derivatives	*Viscum album*	^{13}C and ^{1}H 1D and 2D NMR; HPLC-UV-MS	[74]
**3-(3'-carbomethoxypropyl) -7-3"-protocatechoyl galloate**	Hydroxybenzoic acid derivatives	*Viscum album*	^{13}C and ^{1}H 1D and 2D NMR; HPLC-UV-MS	[74]
**3-(4-hydroxy-3,5-dimethoxy) -phenyl-2E-propenyl-β-D-glucopyranoside**	Hydroxycinnamic acid derivatives	*Viscum album*	IR, UV, HPLC, HR-FAB-MS, ^{1}H- and ^{13}C-NMR.	[45]
**3-(4-acetoxy-3,5-dimethoxy) -phenyl-2E-propenyl-β-D-glucopyranoside**	Hydroxycinnamic acid derivatives	*Viscum album*	IR, UV, HPLC, HR-FAB-MS, ^{1}H- and ^{13}C-NMR.	[45]
**3-OH-cinnamic acid (syn. *m*-coumaric acid)**	Hydroxycinnamic acid derivatives	*Viscum album*	LC-ESI-MS/MS	[39]
**Alangilignoside C**	Hydroxybenzoic acid derivatives - lignan	*Viscum album*	TLC, HPLC-PDA-MS, UFLC-PDA-MS/MS, UHPLC-HRMS/MS	[35]
**Caffeic acid**	Hydroxycinnamic acid derivatives	*Viscum cruciatum, Viscum album*	LC-ESI-MS/MS	[23,97]
**Chlorogenic acid**	Hydroxycinnamic acid derivatives	*Viscum album, Viscum capitellatum, Viscum schimperi, Viscum combreticola*	TLC, UV, NMR, HPLC-PDA-MS, UFLC-PDA-MS/MS, UHPLC-HRMS/MS, UPLC- PDA-ESI-MS/MS	[7,35,72,83,97,116,125,137,138]
**Coniferin (syn. coniferylalcohol 4-O-β-D-glucopyranoside)**	Hydroxycinnamic acid derivatives	*Viscum album*	^{1}H and ^{13}C-NMR, HPLC-MS, HPLC-UV, CG-FID, UV-Vis, TLC, HPLC, UV, IR, and FAB-MS	[21,84]

Table S5. Continued.

Identified compounds	Chemical group	Species	Analytical method	Author(s)
**Phenolic acids and their derivatives – Hydroxycinnamic and Hydroxybenzoic acid derivatives**				
**Coniferylalcohol 4-*O*-apiosyl (1→2) glucoside (syn. Coniferylalcohol-4'-[apiosyl (1 → 2)] glucoside)**	Hydroxycinnamic acid derivatives	*Viscum album*	^{1}H and ^{13}C-NMR, HPLC-MS, HPLC-UV, CG-FID, UV-Vis, TLC, UV-light, IR-810, CD-J 500C, Gas Liquid Chromatography (GLC)	[21,80]
**Ellagic acid**	Hydroxybenzoic acid derivatives	*Viscum orientale*	UHPLC-LC-MS/MS	[135]
**Ferulic acid**	Hydroxycinnamic acid derivatives	*Viscum album, Viscum articulatum*	HPLC-DAD, LC-ESI-MS/MS	[39,72,83,108]
**Feruloyl quinic acid**	Hydroxycinnamic acid derivatives	*Viscum schimperi*	UPLC- PDA-ESI-MS/MS	[137,138]
**Feruloyl quinic acid-methyl ether**	Hydroxycinnamic acid derivatives	*Viscum schimperi*	UPLC- PDA-ESI-MS/MS	[137,138]
**Gallic acid**	Hydroxybenzoic acid derivatives	*Viscum articulatum, Viscum album, Viscum orientale*	HPLC-DAD, ^{13}C and ^{1}H 1D and 2D NMR, HPLC-UV-MS, LC-ESI-MS/MS	[39,72,74,83,99,108,135]
**Kalopanaxin D**	Hydroxycinnamic acid derivatives	*Viscum album*	HPLC, UV, IR, ^{1}H and ^{13}C-NMR, and FAB-MS	[84]
**Ligalbumoside A**	Hydroxybenzoic acid derivatives	*Viscum album*	TLC, HPLC-PDA-MS, UFLC-PDA-MS/MS, UHPLC-HRMS/MS	[35]
**Lyorinesinol**	Hydroxybenzoic acid derivatives - lignan	*Viscum album*	LC-ESI-MS/MS	[90]
***p*-coumaric acid (syn. (E)-3-(4-hydroxyphenyl)-prop-2-enoic acid)**	Hydroxycinnamic acid derivatives	*Viscum capitellatum, Viscum album*	TLC, UV, NMR, HPLC-DAD, LC-ESI-MS/MS	[39,72,83,116]
***p*-hydroxybenzoic acid (syn. 4-OH-benzoic acid)**	Hydroxybenzoic acid derivatives	*Viscum album*	HPLC-DAD, LC-ESI-MS/MS	[39,72,83]
**Protocatechuic acid**	Hydroxybenzoic acid derivatives	*Viscum album*	HPLC-DAD, LC-ESI-MS/MS	[39,72,83]

Table S5. Continued.

Phenolic acids and their derivatives - Hydroxycinnamic and Hydroxybenzoic acid derivatives				
**Identified compounds**	**Chemical group**	**Species**	**Analytical method**	**Author(s)**
**Rosmarinic acid**	Hydroxycinnamic acid derivatives	*Viscum album, Viscum schimperi*	HPLC-DAD, UPLC- PDA-ESI-MS/MS, LC-ESI-MS/MS	[23,39,72,83,99,138]
**Salicylic acid**	Hydroxybenzoic acid derivatives	*Viscum album*	HPLC-DAD, LC-ESI-MS/MS	[39,72,83]
**Sinapic acid**	Hydroxycinnamic acid derivatives	*Viscum album*	HPLC-DAD, LC-ESI-MS/MS	[39,72,83,97,102]
**Sinapoylquinic acid**	Hydroxycinnamic acid derivatives	*Viscum schimperi*	UPLC- PDA-ESI-MS/MS	[137,138]
**Syringenin 4-*O*-apiosyl-glucoside (syn. Polygalatenoside E; Syringenin 4-O-apiosyl (1→2) glucoside)**	Hydroxycinnamic acid derivatives	*Viscum album*	^{1}H and ^{13}C-NMR, HPLC-MS, HPLC-UV, CG-FID, UV-Vis, TLC, HPLC-PDA-MS, UFLC-PDA-MS/MS, UHPLC-HRMS/MS	[21,35]
**Syringenin 4-O-β-D-glucopyranoside (syn. Syringin; Eleutheroside B)**	Hydroxycinnamic acid derivatives	*Viscum album* *Viscum articulatum*	TLC, HPLC-PDA-MS, UFLC-PDA-MS/MS, UHPLC-HRMS/MS, ^{1}H and ^{13}C-NMR, HPLC-MS, HPLC-UV, CG-FID, UV-Vis, TLC, UV-light, IR-810, CD-J 500C, Gas Liquid Chromatography	[7,21,35,80,84,85,102,131]
**Eleutheroside E**	Hydroxycinnamic acid derivatives – lignan	*Viscum coloratum*	UPLC/Q-TOF-MS	[124]
**Syringic acid**	Hydroxybenzoic acid derivatives	*Viscum album*	HPLC-DAD, LC-ESI-MS/MS	[39,72,83]
***trans*-cinnamic acid**	Hydroxycinnamic acid derivatives	*Viscum album*	HPLC-DAD	[72,83]
**Vanillic acid**	Hydroxybenzoic acid derivatives	*Viscum album, Viscum orientale*	LC-ESI-MS/MS	[39,97,135]
**Veratric acid**	Hydroxybenzoic acid derivatives	*Viscum album*	LC-ESI-MS/MS	[39]

Table S6. Flavonoids and their derivatives in alcoholic *Viscum* sp.

Flavonoids and their derivatives – Chalcones

Identified compounds	Species	Analytical method	Author(s)
**2′,4-dihydroxy-4′,6′-dimethoxy chalcone**	*Viscum album*	TLC densitometric method, UV (366 nm); ATR-IR, ^{1}H and ^{13}C – NMR.	[79]
**2′-hidroxy-4′,6′- dimethoxychalcone-4-O-[apiosyl (1→2)]glucoside**	*Viscum album*	TLC; UV-light, IR-810, CD-J 500C, ^{1}H and ^{13}C-NMR, Gas Liquid Chromatography (GLC), HPLC	[82]
**2′-hydroxy-3,4′,6′-trimethoxychalcone-4-O-glucoside**	*Viscum album*	TLC; UV-light, IR-810; CD-J 500C; ^{1}H and ^{13}C-NMR, Gas Liquid Chromatography (GLC), HPLC	[80,82]
**2′-hydroxy-4′,6′ – dimetoxychalcone-4-*O*-glucoside**	*Viscum album*	TLC, UV-light, IR-810, CD-J 500C, ^{1}H and ^{13}C-NMR, Gas Liquid Chromatography (GLC), HPLC	[82]
**2′-hydroxy-4′,6′-dimethoxychalcone-4-O-[apiosyl (1 → 2)]glucoside**	*Viscum album*	TLC, UV-light, IR-810, CD-J 500C, ^{1}H and ^{13}C-NMR, Gas Liquid Chromatography (GLC), HPLC	[80]
**2′-hydroxy-4′,6-dimethoxychalcone-4-O-glucoside**	*Viscum album*	TLC, UV-light, IR-810, CD-J 500C, ^{1}H and ^{13}C-NMR, Gas Liquid Chromatography (GLC), HPLC	[80]

Flavonoids and their derivatives – Anthocyanins

**Malvidin-3-O-glucoside**	*Viscum cruciatum*	LC-ESI-MS/MS	[23]

Flavonoids and their derivatives – Flavan

Identified compounds	Chemical group	Species	Analytical method	Author(s)
**Luteoliflavan-*O*- [pentosyl hexoside]**	Flavan-*O*-glycosides	*Viscum schimperi*	UPLC- PDA-ESI-MS/MS	[137,138]
**Viscutin III (syn. Viscutin 3)**	Flavans	*Viscum schimperi*	UPLC- PDA-ESI-MS/MS	[137,138]
**Epicatechin gallate**	Flavan-3-ol	*Viscum alrticulatum*	HPLC	[131]

Table S6. Continued

**Flavonoids and their derivatives – Flavanones**				
**(2R)-5,7-Dimethoxyflavanone-4′-*O*-glucoside**	Flavanones	*Viscum album*	TLC, UV-light, IR-810, CD-J 500C, ^{1}H and ^{13}C-NMR, Gas Liquid Chromatography (GLC), HPLC	[80,82]
**(2S) Homoeriodictyol-7-*O*-[apiosyl (1 → 2)] glucoside**	Flavanones	*Viscum album*	TLC, UV-light, IR-810, CD-J 500C, ^{1}H and ^{13}C-NMR, Gas Liquid Chromatography (GLC), HPLC	[80]
**(2S) Homoeriodictyol-7-*O*-glucoside**	Flavanones	*Viscum album*	TLC, UV-light, IR-810, CD-J 500C, ^{1}H and ^{13}C-NMR, Gas Liquid Chromatography (GLC), HPLC	[80]
**(2S) -3′,5,7-Trimethoxyflavanone-4′-O-glucoside**	Flavanones	*Viscum album*	TLC, UV-light, IR-810, CD-J 500C, ^{1}H and ^{13}C-NMR, Gas Liquid Chromatography (GLC), HPLC	[80,82]
**4′,5-dimethoxy-7-hydroxyflavanone**	Flavanones	*Viscum album*	IR, UV, HPLC, HR-FAB-MS, ^{1}H- and ^{13}C-NMR	[45]
**4′-O- [β-*D*-apiosyl (1→2)] -β-*D*-glucopyranosyl] -5-hydroxyl-7-*O*-sinapylflavanone**	Flavanones	*Viscum album*	IR, UV, HPLC, HR-FAB-MS, ^{1}H- and ^{13}C-NMR	[45]
**5,7-dimethoxy-4′-hydroxyflavanone**	Flavanones	*Viscum album*	IR, UV, HPLC, HR-FAB-MS, ^{1}H- and ^{13}C-NMR	[45]
**5,7-dimethoxy-4′-*O*-β-*D* glucopyranoside flavanone**	Flavanones	*Viscum album*	IR, UV, HPLC, HR-FAB-MS, ^{1}H- and ^{13}C-NMR	[45]
**7-*O*-β-*D*-glucopyranosyl eriodictyol**	Flavanones	*Viscum capitellatum*	TLC, UV, NMR	[116]
**Eriodictyol (syn. (2S)-2-(3,4-dihydroxyphenyl)-5,7-dihydroxy-4-chromanone)**	Flavanones	*Viscum 13lbum, Viscum capitellatum, Viscum schimperi*	TLC, UV, NMR, LC-ESI-MS/MS; UPLC-PDA-ESI-MS/MS	[39,116,137,138]
**Eriodictyol hexoside**	Flavanones	*Viscum schimperi*	UPLC- PDA-ESI-MS/MS	[137,138]
**Eriodictyol-7-O-β-D-glucopyranoside**	Flavanones	*Viscum album*	IR, UV, NMR, ESI-MS, HR-ESI-MS, HPLC	[75]
**Homeriodictyol-7-O-β-D-apiofuranosyl (1 → 2) -O-β-D-glucopyranoside**	Flavanones	*Viscum album*	IR, UV, NMR, ESI-MS, HR-ESI-MS, HPLC	[75]
**Homoeriodictyol-7-O-β-D-glucopyranoside**	Flavanones	*Viscum 13lbum, Viscum coloratum*	^{1}H and ^{13}C-NMR, MS, HPLC, IR, UV, ESI-MS, HR-ESI-MS, HPLC	[75,120]

Table S6. Continued.

Identified compounds	Chemical group	Species	Analytical method	Author(s)
**Flavonoids and their derivatives – Flavanones**				
**Homoeriodictyol (syn. Hedt-I)**	Flavanones	*Viscum coloratum*	UHPLC-LC-MS/MS, HPLC-MS	[14,121,123]
**Homoeriodictyol-7-O-β-D-glycoside (syn. Hedt-II)**	Flavanones	*Viscum coloratum*	UHPLC-LC-MS/MS, HPLC-MS	[14,121,123]
**Homoeriodictyol-7-O-β-D-apiose (1→2) -β-D-glycoside (syn. Hedt-III)**	Flavanones	*Viscum coloratum*	UHPLC-LC-MS/MS, HPLC-MS	[14,121,123]
**Homoeriodictyol-7-O-β-D-apiose (1→5)-β-D-apiose (1→2)-β-D-glycoside (syn. Hedt-IV)**	Flavanones	*Viscum coloratum*	UHPLC-LC-MS/MS, HPLC-MS	[14,121]
**Homoeriodictyol-7-O-β-D-glucopyranoside-4′-O-β-D-apioside**	Flavanones	*Viscum album*	IR, UV, NMR, ESI-MS, HR-ESI-MS, HPLC	[75]
**Naringenin**	Flavanones	*Viscum album, Viscum schimperi*	UPLC- PDA-ESI-MS/MS, HPLC-DAD, LC-ESI-MS/MS	[7,39,72,83,102,137,138]
**Naringenin-5-methyl ether**	Flavanones	*Viscum album*	TLC; HPLC-PDA-MS, UFLC-PDA-MS/MS, UHPLC-HRMS/MS	[35]
**Naringin**	Flavanones	*Viscum schimperi*	UPLC- PDA-ESI-MS/MS	[137,138]
**Pinobanksin**	Flavanones	*Viscum album*	HR-ESI-MS	[90]
**Sakuranetin**	Flavanones	*Viscum album*	TLC, HPLC-PDA-MS, UFLC-PDA-MS/MS, UHPLC-HRMS/MS, LC-ESI-MS/MS	[35,39,96,99]
**Trihydroxyflavanone-Di-Me-ether-O-[pentosyl-hexoside]**	Flavanones	*Viscum schimperi*	UPLC- PDA-ESI-MS/MS	[137,138]
**Trihydroxyflavanone-Di-Me-ether-O-hexoside**	Flavanones	*Viscum schimperi*	UPLC- PDA-ESI-MS/MS	[137,138]
**Viscumneoside I**	Flavanones	*Viscum coloratum*	UPLC/Q-TOF-MS	[124]

Table S6. Continued.

**Flavonoids and their derivatives – Flavanonols**				
**Identified compounds**	**Chemical group**	**Species**	**Analytical method**	**Author(s)**
**Taxifolin**	Flavanonols	*Viscum album*	LC-ESI-MS/MS	[39]
**Tetrahydroxyflavan-*O*-β-D hexoside**	Flavanonols	*Viscum schimperi*	UPLC- PDA-ESI-MS/MS	[137,138]
**Flavonoids and their derivatives – Flavones**				
**3′,7-dimethoxyluteolin-4′-O-[apiosyl (1→2)]glucoside**	Flavones	*Viscum album*	TLC, UV-light, IR-810, CD-J 500C, ^{1}H and ^{13}C-NMR, Gas Liquid Chromatography (GLC); HPLC	[80]
**3′,7-dimethoxyluteolin-4′-O-glucoside**	Flavones	*Viscum album*	TLC, UV-light, IR-810, CD-J 500C, ^{1}H and ^{13}C-NMR, Gas Liquid Chromatography (GLC), HPLC	[80]
**Apigenin**	Flavones	*Viscum 15lbum, Viscum articulatum*	HPLC, LC-ESI-MS/MS, TLC – densitometric method	[39,57,92,99,108]
**Chrysin**	Flavones	*Viscum album*	LC-ESI-MS/MS	[39]
**Chrysin-*O*-di-*α*-rhamnopyranoside**	Flavones	*Viscum schimperi*	UPLC- PDA-ESI-MS/MS	[137,138]
**Flavoyadorinin B**	Flavones	*Viscum album*	TLC, UV-light, IR-810, CD-J 500C, ^{1}H and ^{13}C-NMR, Gas Liquid Chromatography (GLC), HPLC	[60,80]
**Homoflavoyadorinin B**	Flavones	*Viscum album*	HPLC, TLC, UV-light, IR-810, CD-J 500C, ^{1}H and ^{13}C-NMR, Gas Liquid Chromatography (GLC), HPLC.	[60,80]
**Liquidamboside**	Flavones	*Viscum album*	IR, UV, NMR, ESI-MS, HR-ESI-MS, HPLC	[75]
**Luteolin**	Flavones	*Viscum album*	LC-ESI-MS/MS	[39,97,99]

Table S6. Continued.

Flavonoids and their derivatives – Flavonols				
**Identified compounds**	**Chemical group**	**Species**	**Analytical method**	**Author(s)**
**3-*O*-Methylquercetin**	Flavonols	*Viscum album*	LC-ESI-MS/MS	[39]
**5,7,4′-trihydroxy-3,3′-dimethoxyflavone (syn. Tddf)**	Flavonols	*Viscum coloratum*	HPLC-MS, UHPLC-LC-MS/MS	[14,121]
**5-hydroxy-3,7,3′-trimethoxyflavone-4′-O-β-*D*-glucoside (syn. Httf)**	Flavonols	*Viscum coloratum*	HPLC-MS	[121]
**7-hydroxy-3,5,6,3′,4′-pentamethoxyflavone (syn. Quercetagetin 3,5,6,3′,4'-pentamethylether)**	Flavonols	*Viscum capitellatum*	TLC, UV, NMR	[116]
**Isorhamnetin**	Flavonols	*Viscum album*	LC-ESI-MS/MS	[39,102]
**Isorhamnetin-3-*O*-apiosyl (1→6) glucosyl-7-*O*-rhamnoside**	Flavonols	*Viscum album*	TLC, UV-light, IR-810, CD-J 500C, ^{1}H and ^{13}C-NMR, Gas Liquid Chromatography (GLC), HPLC	[80]
**Isorhamnetin-3-*O*-glucoside**	Flavonols	*Viscum schimperi*	UPLC- PDA-ESI-MS/MS	[137,138]
**Isorhamnetin-3-*O*-rutinoside**	Flavonols	*Viscum album*	TLC, UV-light, IR-810, CD-J 500C, ^{1}H and ^{13}C-NMR, Gas Liquid Chromatography (GLC), HPLC	[80]
**Isorhamnetin-3-*O*-*β*-*D*-glucoside (syn. Isor)**	Flavonols	*Viscum coloratum*	HPLC-MS, UHPLC-LC-MS/MS	[14,121]
**Isorhamnetin-*O*-pentosyl-*O*-*α*-L-rhamnopyranoside**	Flavonols	*Viscum schimperi*	UPLC- PDA-ESI-MS/MS	[137,138]
**Hyperoside**	Flavonols	*Viscum cruciatum*	LC-ESI-MS/MS	[23]
**Kaempferol**	Flavonols	*Viscum album*<br>*Viscum cruciatum*	HPLC-UV, LC-ESI-MS/MS, HPLC-DAD	[23,39,72,73,78,83]
**Morin**	Flavonols	*Viscum album*	LC-ESI-MS/MS	[39]

Table S6. Continued.

Flavonoids and their derivatives - Flavonols				
**Identified compounds**	**Chemical group**	**Species**	**Analytical method**	**Author(s)**
**Myricetin**	Flavonols	*Viscum album*	LC-ESI-MS/MS	[39]
**Pachypodol (syn. Pach)**	Flavonols	*Viscum coloratum*	HPLC-MS, UHPLC-LC-MS/MS	[14,121]
**Prunetin**	Flavones	*Viscum album*	LC-ESI-MS/MS	[39]
**Quercetin**	Flavonols	*Viscum album, Viscum orientale*	TLC - densitometric method, HPLC-UV, LC-ESI-MS/MS	[39,57,72,73,78,83,90,135]
**Quercitrin**	Flavonols	*Viscum cruciatum*	LC-ESI-MS/MS	[23]
**Rhamnazin**	Flavonols	*Viscum album*	IR, UV, NMR, ESI-MS, HR-ESI-MS, HPLC	[39,75]
**Rhamnazin-3,4'-di-O-glucoside**	Flavonols	*Viscum album*	TLC, UV-light, IR-810, CD-J 500C; ^{1}H and ^{13}C-NMR, Gas Liquid Chromatography (GLC), HPLC	[80]
**Rhamnazin-3-O-β-D-(6′′-acetyl) -O-β-D-glucopyranoside**	Flavonols	*Viscum album*	IR, UV, NMR, ESI-MS, HR-ESI-MS, HPLC	[75]
**Rhamnazin-3-O-β-D-(6''-β-hydroxy-β-methyglutaryl) -glucoside (syn. Rham-II)**	Flavonols	*Viscum coloratum*	HPLC-MS	[121]
**Rhamnazin-3-O-β-D-glucopyranoside**	Flavonols	*Viscum album*	IR, UV, NMR, ESI-MS, HR-ESI-MS, HPLC	[75]
**Rhamnazin-3-O-β-D-glucoside (syn. Rham-I)**	Flavonols	*Viscum coloratum*	HPLC-MS	[121]
**Rhamnazine-3-O-β-D-apiofuranosyl (1→2) -[6''-(3-oxhydryl-3-methylgluaryl)] -O-β-D-glucopyranoside**	Flavonols	*Viscum album*	IR, UV, NMR, ESI-MS, HR-ESI-MS, HPLC	[75]
**Rhamnetin**	Flavonols	*Viscum album*	LC-ESI-MS/MS	[39]
**Rutin**	Flavonols	*Viscum album*	HPLC	[73,78]

Table S7. Terpenoids and their derivatives in alcoholic *Viscum* sp.

**Terpenoids and their derivatives**				
**Identified compounds**	**Chemical group**	**Species**	**Analytical method**	**Author(s)**
**(1 R,7S)-1,12,13-trihydroxybisabola-3,10-diene**	Sesquiterpene	*Viscum coloratum*	^{13}C NMR, HR-ESI-MS	[122]
**Roseoside**	Sesquiterpene	*Viscum album*	HPLC-MS/MS	[102]
**3-epi-betulinic acid**	Pentacyclic triterpenoid derivatives	*Viscum coloratum*	TLC, ^{13}C and ^{1}H NMR, ESI-MS	[118]
**Betulin (syn. 3β-lup-20(29)-en-3,28-diol)**	Pentacyclic triterpenoid derivatives	*Viscum capitellatum, Viscum album*	TLC, UV, NMR, HPLC-UV	[52,116]
**Betulinic acid (syn. 3β-hydroxy-lup-20(29)-en-28-oic acid, betulic acid)**	Pentacyclic triterpenoid derivatives	*Viscum album, Viscum angulatum, Viscum articulatum, Viscum capitellatum, Viscum schimperi*	TLC, UV, UPLC- PDA-ESI-MS/MS, HPLC-UV, HPTLC, HPLC, UV-light, IR-810, CD-J 500C, ^{1}H and ^{13}C-NMR, Gas Liquid Chromatography (GLC)	[52,72,80,82,83,96,105,112,116,137,138]
**Betulonic acid (syn. Liquidambaric acid)**	Pentacyclic triterpenoid derivatives	*Viscum coloratum*	TLC, ^{13}C and ^{1}H NMR, ESI-MS	[118]
**Campesterol**	Steroid derivatives	*Viscum album*	GC-MS	[40]
**Carotenoids**	Tetraterpenoids	*Viscum capitellatum*	TLC, UV, NMR	[116]
**Lupeol (syn. lup-20(29)-en-3β-ol)**	Pentacyclic triterpenoid derivatives	*Viscum album, Viscum capitellatum*	TLC, UV, NMR, GC-MS	[40,116]

Table S7. Continued.

Terpenoids and their derivatives				
**Identified compounds**	**Chemical group**	**Species**	**Analytical method**	**Author(s)**
**Oleanolic acid (syn. 3β-hydroxyolean-12-en-28-oic acid)**	Pentacyclic triterpenoid derivatives	*Viscum album, Viscum angulatum, Viscum articulatum, Viscum capitellatum, Viscum coloratum, Viscum schimperi*	TLC, UV, HPTLC, HPLC, UPLC- PDA-ESI-MS/MS, ESI-MS, TLC; UV-light, IR-810, CD-J 500C, ^{1}H and ^{13}C-NMR, Gas Liquid Chromatography (GLC)	[80,82,96,105,108,112–114,116,118,137,138]
**Ursolic acid**	Pentacyclic triterpenoid derivatives	*Viscum articulatum*	HPLC	[131]
**phytosterol**	Phytosterols	*Viscum album*	TLC, UV-light, IR-810, CD-J 500C, ^{1}H and ^{13}C-NMR, Gas Liquid Chromatography (GLC), HPLC	[80,82]
**phytosterol-glycoside**	Phytosterols	*Viscum album*	TLC, UV-light, IR-810, CD-J 500C, ^{1}H and ^{13}C-NMR, Gas Liquid Chromatography (GLC), HPLC	[80]
**phytosterol-β-D-glucoside**	Phytosterols	*Viscum album*	TLC, UV-light, IR-810, CD-J 500C, ^{1}H and ^{13}C-NMR, Gas Liquid Chromatography (GLC), HPLC	[82]
**Spinasterol (syn. stigmasta-7,22-dien-3β-ol)**	Phytosterols	*Viscum capitellatum*	TLC, UV, NMR	[116]
**α-tocopherol**	Tocopherols	*Viscum album*	GC-MS	[40]
**β-amyrin acetate**	Triterpenoids	*Viscum album, Viscum cruciatum, Viscum coloratum*	TLC, UV, FAB-Mass, ^{13}C and ^{1}H NMR, TLC, 13C and 1H NMR, ESI-MS, TLC, UV-light, IR-810, CD-J 500C, Gas Liquid Chromatography (GLC), HPLC	[80,82,118,127]
**β-amyrin**	Triterpenoids	*Viscum album*	GC-MS	[40]
**β-sitosterol (syn. stigmast-5-en-3-ol)**	Phytosterols	*Viscum album, Viscum capitellatum*	TLC; UV; NMR; GC-MS	[40,116]

Table S8. Miscelaneous in alcoholic *Viscum* sp.

Identified compounds	Chemical group	Species	Analytical method	Author(s)
**Miscelaneous**				
**Capsaicin**	Methoxyphenols	*Viscum articulatum*	HPLC	[108]
**Lapachol**	Naphtoquinones	*Viscum articulatum*	HPLC	[108]
**Quinic acid**	Cyclitol	*Viscum schimperi, Viscum album*	UPLC- PDA-ESI-MS/MS	[90,96,137,138]
**2,4-heptadienal**	Aldehydes	*Viscum album*	GC–MS	[33]
**3-methylsilane**	Silanes	*Viscum album*	GC–MS	[33]
**2-methylfuran**	Furans	*Viscum album*	GC–MS	[33]
**1,7-Di-(3′,4′ dihydroxyphenyl)-4-hepten-3-one**	Diarylheptanoids	*Viscum cruciatum*	IR; ^{13}C and ^{1}H NMR, HR-MS	[128]
**1 R,2S,3S,5S)-2,3-dihydroxy-3′,3′′-dimethoxy-4′-de-O-methylcentrolobine**	Diarylheptanoids	*Viscum coloratum*	^{13}C NMR, HR-ESI-MS	[122]
**(2Z, 6Z)-dibutyl octa-2, 6-dien-4-yne dioate**	Unsaturated organic acids	*Viscum album*	IR, UV, NMR, ESI-MS, HR-ESI-MS, HPLC	[75]
**(2E, 6E)-dibutyl octa-2, 6-dien-4-yne dioate**	Unsaturated organic acids	*Viscum album*	IR, UV, NMR, ESI-MS, HR-ESI-MS, HPLC	[75]
**Arginine**	Amino acid	*Viscum album*	UPLC-ESI-MS/MS	[7]
**Glutamic acid**	Amino acid	*Viscum album*	UPLC-ESI-MS/MS	[7]
**Pinitol**	Cyclitol	*Viscum album*	UPLC-ESI-MS/MS	[7]
**Sinapyl alcohol**	Monolignol	*Viscum album*	UPLC-ESI-MS/MS	[7]
**Volatile compounds**				
**166 substances**	53 esters; 32 alcohols; 25 terpenes; 18 carbonyl compounds, such as 12 aldehydes and 6 ketones; 12 alkanes; 9 acetals; 8 acids and; 9 other (undefined) organic compounds	*Viscum album*	GC-MS	[86]
